# Loneliness as a Predictor of Blunted Heart Rate Variability Responses During Cognitive Demands

**DOI:** 10.1111/psyp.70432

**Published:** 2026-11-13

**Authors:** Charlotte M. Roddick, Yuxiao Wang, Jiaying Zhao, Lorne A. Whitehead, Jennifer A. Veitch, Michael P. Royer, Dorukalp Durmus, Frances S. Chen

**Affiliations:** ^1^ University of British Columbia Vancouver British Columbia Canada; ^2^ National Research Council of Canada Ottawa Ontario Canada; ^3^ Pacific Northwest National Laboratory Portland Oregon USA; ^4^ Pennsylvania State University University Park Pennsylvania USA

## Abstract

Loneliness has been linked to adverse health outcomes, yet little is known about how different temporal dimensions of loneliness—chronic and situational—relate to physiological flexibility during cognitive demands. This study examined whether chronic loneliness (past 5 years) and situational loneliness (past 24 h) predicted vagally mediated heart rate variability (vmHRV) reactivity across diverse cognitive tasks. Participants (*N* = 151) completed attention, perception, short‐term memory, working memory, and executive function tasks while continuous electrocardiography was recorded. Linear mixed‐effects models revealed that higher chronic loneliness was robustly associated with blunted vmHRV reactivity across attention, perception, working memory, and executive function demands, indicating reduced cardiac vagal withdrawal. These associations persisted after adjusting for differences in depressive symptoms, anxiety, perceived stress, emotion regulation, structural social network characteristics, health behaviors, and demographic factors. Although situational loneliness showed weaker and less consistent associations, exploratory analyses suggested that concurrent elevations in chronic and situational loneliness may be linked to especially blunted vmHRV reactivity during working memory demands, supporting a potential additive burden of loneliness. These findings underscore the value of distinguishing between temporal dimensions of loneliness and highlight chronic loneliness as an independent correlate of disrupted neurovisceral regulation. Addressing persistent feelings of social disconnection may be important not only for psychological well‐being but also for maintaining physiological systems that support adaptive cognitive functioning and long‐term health.

## Impact Statement

Chronic loneliness is often treated as a psychological concern, yet our findings show it is also closely tied to physiological flexibility during everyday cognitive demands. We demonstrate that chronic—but not transient—loneliness is associated with blunted cardiac vagal withdrawal across diverse cognitive challenges. This pattern identifies chronic loneliness as a distinct correlate of diminished neurovisceral flexibility, offering a new mechanistic perspective on loneliness‐related health risk.

## 1 Introduction

Loneliness is a recognized risk factor for adverse health outcomes, including cardiovascular disease and mortality (Card et al. 2022; Hawkley and Cacioppo 2010; Holt‐Lunstad et al. 2015). Dysregulated cardiac parasympathetic functioning—indexed by vagally mediated heart rate variability (vmHRV)—may represent a potential mechanism linking loneliness to disrupted physiological processes. More broadly, blunted cardiovascular reactivity to psychological stress—reflecting smaller physiological changes in response to environmental demands—has been prospectively associated with adverse cardiovascular outcomes such as myocardial infarction and carotid atherosclerosis (O'Riordan et al. 2023), suggesting that diminished cardiovascular responsiveness to environmental demands may represent a pathway to poorer health. In line with this possibility, research has found that lonelier individuals exhibit blunted vmHRV reactivity during cognitive demand in the form of a visual attention task (Muhtadie et al. 2015; Roddick and Chen 2021). However, it remains unknown whether the effects of loneliness on vmHRV reactivity generalize to other cognitive challenge tasks (i.e., tasks designed to elicit demands on cognitive processes such as attention and working memory, as opposed to primarily provoking overt stress responses; Epel et al. 2018). It is also unknown how the duration of loneliness may influence vmHRV responses during cognitive demands. Different cognitive processes engage partially distinct neural networks and may impose varying demands on autonomic regulation (McIntosh et al. 2020; Schmaußer et al. 2024; Tau and Peterson 2010), making it important to clarify whether loneliness disrupts cardiac vagal regulation broadly or in ways that are specific to particular types of cognitive load.

### 1.1 Loneliness and vmHRV Reactivity During Cognitive Demands

Although loneliness is linked to impaired cognitive functioning, the underlying neuropsychological mechanisms and the specificity of these effects remain unclear (Wong et al. 2022). In older adults, loneliness has been associated with both process‐specific deficits (e.g., working memory, visuospatial function) and broader cognitive impairments, including accelerated declines in global cognitive and executive functioning (Harrington et al. 2023; Samtani et al. 2022; Sundström et al. 2020). These observations raise the question of whether loneliness disproportionately affects particular cognitive processes—such as attentional control within frontoparietal networks—or may contribute to more pervasive disruptions across multiple cognitive processes (Jin et al. 2025; Wong et al. 2022). Although overt differences in cognitive performance may not yet be evident among younger adults, loneliness‐related dysregulation may be detectable at a neurophysiological level (Hawkley and Capitanio 2015; Morr et al. 2022).

The neurovisceral integration model (Thayer and Lane 2009) provides a conceptual framework for understanding how loneliness‐related cognitive disruptions may manifest peripherally in altered cardiac vagal regulation. According to this model, cognitive control and autonomic regulation—including vmHRV reactivity—are coordinated by a distributed neural network that includes the medial prefrontal cortex (mPFC), anterior cingulate cortex (ACC), insula, and amygdala (Beauchaine 2015; Nguyen et al. 2022; Thayer and Lane 2009). Within this “central autonomic network,” the mPFC plays a critical role in exerting top‐down inhibitory control over subcortical structures, regulating both cognitive processes and autonomic responses (Beauchaine 2015; Thayer et al. 2009). During cognitively demanding tasks, decreases in vmHRV (i.e., vagal withdrawal) are thought to reflect task engagement and adaptive shifts in attention that support cognitive functioning (Schmaußer et al. 2024; Thayer and Lane 2000, 2009). Conversely, blunted vmHRV reactivity—smaller decreases in vmHRV from baseline to task—may indicate disruptions in cognitive‐autonomic integration, suggesting diminished capacity to flexibly modulate autonomic responses to meet cognitive demands (Carroll et al. 2017; Nguyen et al. 2022). In younger adults, loneliness may be associated with this underlying pattern of dysregulation in cognitive‐autonomic (i.e., neurovisceral) integration, even in the absence of overt cognitive performance deficits.

The health relevance of blunted cardiovascular reactivity may lie partly in what it reflects about these shared brain–body regulatory systems. Blunted physiological responses to environmental demands may indicate altered functioning of neural regions that coordinate both cardiovascular responding and adaptive behavior, including the mPFC, ACC, insula, and amygdala (Carroll et al. 2017). As a result, attenuated cardiovascular reactivity may serve as a peripheral marker of broader neurovisceral dysregulation, offering a possible explanation for why blunted cardiovascular reactivity has been linked to poorer prospective health outcomes (O'Riordan et al. 2023).

Emerging evidence supports a link between loneliness and altered physiological reactivity during acute laboratory challenge contexts (Brown et al. 2018). More specifically, in two studies examining cognitive challenge, chronic loneliness has been associated with blunted vmHRV reactivity during a multiple object tracking task that places substantial demands on visuospatial attention (Muhtadie et al. 2015; Roddick and Chen 2021). It is not yet known, however, whether this effect is specific to attentional control or may extend to other cognitive processes, such as perception, short‐term and working memory, and executive function. Determining whether loneliness‐related vmHRV blunting is domain‐general or domain‐specific may help clarify which components of cognitive‐autonomic integration may be most affected by loneliness. If loneliness is broadly associated with blunted vmHRV reactivity across diverse cognitive demands, it could suggest a potentially pervasive disruption of neurovisceral integration that may heighten vulnerability to cognitive‐affective difficulties (Thayer and Lane 2009). By contrast, if the effects of loneliness are limited to particular cognitive processes, this may suggest more targeted disruptions in cognitive‐autonomic regulation. To address these possibilities, this study examines vmHRV reactivity across multiple cognitive challenge tasks, analyzed separately to assess potential domain‐specific effects of loneliness. This approach provides a nuanced examination of the neurovisceral integration model and the potential role of vmHRV as a physiological indicator of loneliness‐related dysregulation.

Outside of controlled experimental tasks, “real‐world” cognitive functioning rarely involves isolated processes. Most daily activities require the simultaneous engagement of cognitive functions such as attention, perception, memory, and executive skills. If loneliness broadly impairs vagal regulation across these processes, this physiological dysregulation could potentially lead to compromised decision‐making, academic and workplace performance, and overall cognitive efficiency (Cristofori et al. 2019). From an applied perspective, cross‐domain vagal dysregulation may not only impair day‐to‐day cognitive functioning but also contribute to longer‐term health risks—further underscoring the importance of addressing loneliness as a modifiable risk factor for cognitive and cardiovascular decline (Jin et al. 2025).

### 1.2 Temporal Dimensions of Loneliness

Furthermore, the temporal dimensions of loneliness may shape its physiological consequences. Evolutionary perspectives about the function of loneliness propose that loneliness can trigger vigilance for threats and motivate individuals to restore social bonds (Cacioppo et al. 2014). However, when experienced chronically, loneliness can drive sustained hypervigilance and repeated activation of stress‐regulatory systems, eventually contributing to cumulative physiological wear and tear, or “social allostatic load” (Quadt et al. 2020).

Conceptual frameworks have distinguished between chronic and more transient forms of loneliness, reflecting differences in duration and potential underlying mechanisms (Marangoni and Ickes 1989; Young 1982). Although there is no universally agreed‐upon threshold for these durations (Wolska and Creaven 2023), chronic loneliness is typically understood as a prolonged experience persisting over years, linked to stable dispositional or cognitive patterns (Vanhalst et al. 2015, 2018; Young 1982), whereas situational loneliness refers to more transient feelings arising across a single day or in response to specific social contexts (Doane and Adam 2010; Hall 2025). Individual studies have variously operationalized chronic loneliness as persisting for 1–6 years (e.g., Cutrona 1982; Martín‐María et al. 2021; Ojagbemi et al. 2021; Shiovitz‐Ezra and Ayalon 2010; Theeke 2010; Vanhalst et al. 2018), whereas situational loneliness has been assessed in relation to day‐level fluctuations or discrete events (Doane and Adam 2010; Saporta et al. 2021).

Loneliness, as measured by versions of the UCLA scale (Russell et al. 1980; Russell 1996), which is generally considered a measure of chronic loneliness (Marangoni and Ickes 1989; Quadt et al. 2020), has been linked to both blunted (heart rate, cardiac output) and exaggerated (blood pressure, inflammatory markers) cardiovascular reactivity to acute stress, with blunted patterns often associated with impaired vmHRV withdrawal (Brindle et al. 2014; Brown et al. 2018; Carroll et al. 2017). Such blunted cardiovascular reactivity is thought to reflect insufficient engagement of fronto‐limbic circuits essential for autonomic regulation and motivated behavior (Carroll et al. 2017; Gianaros et al. 2012; Ginty et al. 2013). Given that loneliness is also linked to maladaptive emotion regulation and motivational deficits (Preece et al. 2021; Spithoven et al. 2017; Vanhalst et al. 2018), the effects of chronic loneliness on cardiac vagal regulation may plausibly manifest as blunted vmHRV reactivity to cognitive or emotional challenges. As such, chronic loneliness—persisting over several years—could predict a generalized pattern of blunted vmHRV reactivity to cognitive challenges, similar to the pattern previously observed during demands on visuospatial attention (Muhtadie et al. 2015; Roddick and Chen 2021).

By contrast, situational loneliness—experienced more transiently, such as over the course of a single day (Doane and Adam 2010)—may function as an adaptive signal that motivates social reconnection efforts, akin to hunger or thirst signaling an unmet need (Cacioppo et al. 2015). From this perspective, situational loneliness could mobilize physiological systems in preparation for social engagement or increased metabolic demands, potentially manifesting as heightened vmHRV reactivity to cognitive challenges (Porges 2007; Qualter et al. 2015). Alternatively, situational loneliness may elicit blunted vmHRV reactivity, but with smaller effects than those expected for chronic loneliness, given situational loneliness lacks the prolonged physiological embedding suggested to characterize chronic loneliness (Matthews and Tye 2019; Quadt et al. 2020). Thus, whereas chronic loneliness may embed maladaptive patterns of vagal dysregulation (Quadt et al. 2020), situational experiences of loneliness could plausibly lead to either exaggerated or blunted vmHRV reactivity, depending on whether situational loneliness primarily activates short‐term affiliative processes or reflects early signs of regulatory strain. Chronic and situational loneliness may also interact; for example, individuals reporting both high chronic and high situational loneliness may exhibit particularly blunted vmHRV responses across cognitive challenges.

### 1.3 Research Questions and Hypotheses

To investigate these questions, we examined the effects of chronic and situational loneliness on vmHRV reactivity across multiple cognitive challenge tasks. Chronic loneliness was assessed as self‐reported feelings of loneliness over the past 5 years, whereas situational loneliness was measured as self‐reported loneliness over the preceding 24 h. By adopting a 5‐year timeframe to assess chronic loneliness and a 24‐h window to capture situational loneliness, this study builds on prior conceptualizations of chronic loneliness as an enduring phenomenon persisting over years (Young 1982; Wolska and Creaven 2023) and situational loneliness as more transient daily fluctuations (Doane and Adam 2010), while also ensuring these constructs are temporally distinct. VmHRV reactivity was evaluated across tasks targeting diverse cognitive processes, specifically: attention, perception, short‐term memory, working memory, and executive function.

The primary aim of this study was to assess how temporal dimensions of loneliness—chronic and situational—affect vmHRV reactivity during diverse cognitive challenges (i.e., demands placed on cognitive function). Additionally, we conducted exploratory analyses to examine whether chronic and situational loneliness interact to influence vmHRV reactivity.

*H1 (chronic loneliness)*. Greater chronic loneliness—as experienced over the past 5 years—was hypothesized to predict blunted vmHRV reactivity across multiple cognitive challenge tasks.

*H2 (situational loneliness)*. Greater situational loneliness—as experienced over the past 24 h—was hypothesized to predict altered vmHRV responses to cognitive challenge tasks. Based on competing theoretical perspectives and limited empirical work addressing how situational loneliness affects physiological responses, this effect could plausibly manifest as either exaggerated or blunted vmHRV reactivity (Doane and Adam 2010; Porges 2007; Matthews and Tye 2019; Quadt et al. 2020). Therefore, no directional hypothesis was specified.

*Exploratory (interaction effects)*. We also explored whether chronic and situational loneliness interact, with situational loneliness potentially moderating the effects of chronic loneliness on vmHRV reactivity to the cognitive challenge tasks. Given the limited existing empirical work and the theoretical ambiguity surrounding the main effects of situational loneliness, these analyses were considered exploratory.

## 2 Methods

### 2.1 Participants

Participants were undergraduate students recruited for a study on “indoor environments” from The University of British Columbia's Human Subject Pool between January and April 2022. Exclusion criteria included having a chronic medical condition, cardioactive medication use (e.g., antidepressants, beta‐blockers), self‐identification as a smoker, color‐deficient vision, lack of normal or corrected‐to‐normal vision, and non‐proficiency in English. Of the 156 participants enrolled, five female participants were excluded from analyses: two due to incomplete sessions,^1^ two for cardioactive medication use, and one due to corrupted ECG data. The final analytic sample comprised 151 participants (117 females, 34 males; mean age = 20.30 years, SD = 2.70; range = 16–44 years; see Table 1 for sample descriptives). Ethical approval for the study was granted by the university's Behavioral Research Ethics Board, and all participants provided written informed consent prior to enrollment.

**TABLE 1:** Sample descriptives.

	*M* (SD) or %
Demographics	
Age	20.30 (2.70)
Body mass index (BMI)	22.49 (3.97)
Female	77.5%
Health behaviors	
Hours slept the previous night	7.10 (1.50)
Average hours of sleep	7.28 (1.08)
Average hours of weekly exercise	3.41 (3.70)
Drink alcohol once a month or less	71.5%
Drink caffeine once a week or less	41.1%

	*M* (SD)	Possible range
Psychosocial measures		
Chronic loneliness (modified UCLA: 5 years)	44.00 (10.49)	[20–80]
Situational loneliness (modified UCLA: 24 h)	37.82 (11.32)	[20–80]
Depressive symptoms (CESD)	15.41 (9.60)	[0–60]
Anxiety symptoms (BAI)	18.28 (14.50)	[0–99]
Perceived stress (PSS)	17.68 (6.58)	[0–40]
Emotion regulation: cognitive reappraisal (ERQ)	29.30 (6.51)	[6–42]
Emotion regulation: expressive suppression (ERQ)	15.18 (5.57)	[4–28]
Social network size (SNI)	23.81 (23.20)	[0–∞]
Social network diversity (SNI)	6.01 (1.29)	[0–12]

*Note: N* = 151. Ethnicity: The most commonly reported ethnic background was East Asian (e.g., Chinese, Japanese, or Korean; 40.7%), followed by Caucasian (21.9%), South Asian (e.g., Indian; 14.6%), Mixed (9.9%), Southeast Asian (e.g., Vietnamese, Filipino; 5.3%), Hispanic/Latino (4.0%), African (4.0%), and Middle Eastern (1.3%), with one participant identifying their ethnicity as Other (0.7%). Minor demographic differences from the prior project on near‐infrared lighting (Roddick et al. 2024) reflect slight differences in sample composition.

Abbreviations: BAI, Burns Anxiety Inventory; CESD, Center for Epidemiological Studies Depression; ERQ, Emotion Regulation Questionnaire; PSS, Perceived Stress Scale; SNI, Social Network Index; UCLA, UCLA Loneliness Scale.

### 2.2 Procedure

As part of a broader research protocol, participants individually attended a 2‐h laboratory session during which their vmHRV was recorded at rest and during several cognitive challenge tasks. Participants were instructed to refrain from vigorous exercise and from consuming food, caffeine, or alcohol for 3 h prior to the session. At the start of the session, participants were fitted with an ECG sensor and electrodes in a modified lead II configuration. Participants subsequently remained seated for an adaptation period (~30 min) while completing self‐report questionnaires, allowing time to acclimate to the laboratory environment (Laborde et al. 2017).

Following the adaptation period, participants were guided to a study cubicle where they completed a sequence of rest periods and cognitive tasks. The session was structured into two sequential blocks, each lasting approximately 45 min. Within each block, participants first completed a 5‐min rest period, followed by a neutrally valenced task^2^ not relevant to the current study (~10 min), another 5‐min rest, and then a series of cognitive tasks assessing attention, perception, short‐term and working memory, and executive function (~15 min). A final 5‐min rest period concluded the block. After the first block, participants left the cubicle for a short break (~4 min) in another area of the laboratory before returning to complete the second block. Throughout each block, vmHRV was continuously recorded via ECG, and participants remained seated. At the end of the session, height and weight were measured, and participants were debriefed. The present study focuses on the portions of the procedure involving vmHRV measurement during the rest periods at the start of each block and the subsequent cognitive tasks (see supplemental Figure S1 for a visual depiction of the study procedure).

### 2.3 Measures

#### 2.3.1 Chronic and Situational Loneliness

Chronic and situational loneliness were assessed using two modified versions of the UCLA Loneliness Scale (Version 3; Russell 1996), a validated and widely used measure consisting of 20 items, such as “How often do you feel close to people?” and “How often do you feel alone?” The original scale captures general perceptions of loneliness without referencing a specific timeframe (Marangoni and Ickes 1989). In this study, each item was presented once and followed by two timeframes. Participants were asked to rate how often they felt the way described in each item “over the past 24 h” (situational loneliness) as well as “over the past five years” (chronic loneliness). For example, participants were asked, “How often do you feel alone…” with the response options on a 4‐point scale from 1 (never) to 4 (often) provided separately for each timeframe. Internal consistency was excellent for both chronic (Cronbach's *α* = 0.93) and situational (Cronbach's *α* = 0.93) loneliness. Chronic and situational loneliness were moderately positively correlated, *r* = 0.38, *p* < 0.001 (see Table S1 for correlations between psychological measures).

#### 2.3.2 Vagally‐Mediated Heart Rate Variability (vmHRV)

The selection and implementation of all equipment and procedures were derived from HRV research guidelines (Berntson et al. 1997; Laborde et al. 2017; Quigley et al. 2024). VmHRV was continuously recorded using the Bittium Faros 180 electrocardiogram (ECG) system at a sampling rate of 1000 Hz. Disposable Ag/AgCl gel electrodes were placed in a modified Lead II configuration (i.e., right clavicle, left lower torso). The digitized ECG signal was extracted and analyzed in 60‐s epochs using Kubios HRV Premium (version 3.5). Artifact detection and correction followed established guidelines for HRV research (Berntson et al. 1997; Laborde et al. 2017). Automated artifact detection (Kubios HRV 3.5; Tarvainen et al. 2021) was supplemented with visual inspection, and corrections were applied where necessary. ECG data were excluded if over 5% of beats required correction, if artifacts were visually identified as excessive, or if the data contained physiologically implausible values.

VmHRV was quantified using the root mean square of successive differences (RMSSD, ms), a widely used time‐domain index of cardiac vagal modulation considered less susceptible to respiratory influences than frequency‐domain measures (Berntson et al. 2005; Hill and Siebenbrock 2009; Laborde et al. 2017). Sensitivity analyses were also conducted using high‐frequency HRV (HF‐HRV, ln ms^2^), a commonly used frequency‐domain indicator of cardiac vagal function, as a secondary metric (see Supporting Information). The HF‐HRV data were detrended using the smoothness priors method (Tarvainen et al. 2002) and submitted to a Fast Fourier Transform (FFT) to determine spectral power values (in ms^2^/Hz). RMSSD and HF‐HRV were selected as they are validated indices of cardiac vagal regulation and the most frequently reported in the literature, facilitating comparability across studies (Laborde et al. 2017; Roddick et al. 2025). VmHRV reactivity was defined as the change in vmHRV between the resting baseline and each cognitive challenge task.

#### 2.3.3 Cognitive Challenge Tasks

Participants completed a series of timed cognitive challenge tasks, each designed to elicit vmHRV reactivity during demands on one of five distinct cognitive processes: attention, perception, short‐term memory, working memory, and executive function. The tasks were administered twice, once during each of the two parallel blocks of the procedure. Although the same types of tasks were administered in both blocks, different items of equivalent difficulty were used to minimize practice effects. Descriptions and example trials for each task, including participant instructions, are provided in Supporting Information.

##### 2.3.3.1 Attention

Attention was assessed using a visual search task employing a foraging paradigm to measure top‐down attentional control (Wolfe 2012, 2013). Participants completed two trials of the task, each lasting 90 s. In each trial, participants searched for 50 target stimuli (rotated capital letter Ts) embedded among 500 distractors (capital letter Ls), randomly distributed within a 16 cm × 16 cm square. Targets and distractors were presented in size 7 Arial font, each measuring 0.125 cm in height and 0.25 cm in width. Participants were instructed to locate and circle as many targets as possible within the allotted time. Attentional performance was measured by the total number of targets correctly identified across both trials.

##### 2.3.3.2 Perception

Perception was evaluated using a visual comparison task designed to assess the ability to detect subtle differences between paired images (Manns et al. 2000). This task measures change detection failure, which can be influenced by attentional limitations or working memory constraints (Rensink 2009). Participants completed two trials of the task, with each trial lasting 2 min. In each trial, participants were presented with two nearly identical images displayed side by side and were instructed to identify and circle as many differences as possible. The sum of correctly identified differences across both trials was used to assess perceptual performance.

##### 2.3.3.3 Short‐Term and Working Memory

A digit span task was used to assess short‐term and working memory (Diamond 2013; Kane et al. 2004). Participants listened to pre‐recorded sequences of digits and were required to recall them verbally in either the same order (short‐term memory) or in reverse order (working memory). Short‐term memory was measured using 10 forward span trials, with sequence lengths increasing from 3 to 12 digits. Working memory was assessed using six backward span trials, with sequence lengths increasing from three to eight digits. Performance for short‐term memory and for working memory was each measured by the total number of correctly recalled trials, as well as by the number of digits correctly recalled in the last successfully completed trial.

##### 2.3.3.4 Executive Function

Executive function was assessed using a flanker task designed to evaluate participants' ability to suppress non‐relevant, conflicting information while identifying the direction of a central target stimulus (Eriksen and Eriksen 1974; Mullane et al. 2009). Each participant completed 100 trials in which they were presented with five arrows displayed in a row. The central arrow pointed either left or right, and participants were instructed to indicate its direction by circling “L” for left or “R” for right, while ignoring the surrounding arrows. Performance was measured as the total number of correct responses across all trials.

#### 2.3.4 Psychosocial Measures

In addition to assessing chronic and situational loneliness, several psychological factors were measured using validated scales and included as potential covariates in analyses. These included depressive symptoms, anxiety, perceived stress, emotion regulation, and social network size and diversity. Depressive symptoms were measured using the Center for Epidemiologic Studies Depression Scale (CES‐D; Radloff 1977). To avoid conceptual overlap with the loneliness measures, the item “I felt lonely” was not included, resulting in a 19‐item version of the CES‐D (Vanhalst et al. 2018). Internal consistency for the 19‐item CES‐D was excellent (Cronbach's *α* = 0.91). Anxiety was assessed using the 33‐item Burns Anxiety Inventory (BAI; Burns and Eidelson 1998; Cronbach's *α* = 0.94), and perceived stress was measured with the 10‐item Perceived Stress Scale (PSS; Cohen and Williamson 1988; Cronbach's *α* = 0.90). Emotion regulation was assessed using the cognitive reappraisal and expressive suppression subscales of the 10‐item Emotion Regulation Questionnaire (ERQ; Gross and John 2003; reappraisal *α* = 0.85, suppression *α* = 0.80). Social network size and social network diversity were measured using the corresponding dimensions of the 13‐item Social Network Index^3^ (SNI; Cohen et al. 1997). See Table S1 for correlations between psychological measures.

#### 2.3.5 Demographics and Health Behaviors

In line with best practice guidelines for vmHRV research (Berntson et al. 1997; Laborde et al. 2017; Quigley et al. 2024), demographic and health behavior variables commonly recommended as covariates were collected to account for potential confounding influences. Participants self‐reported their age, sex,^4^ hours slept the previous night, average hours of sleep per night, exercise frequency, and typical caffeine and alcohol consumption. Body mass index (BMI) was calculated from measured height and weight.

### 2.4 Declaration of Generative Artificial Intelligence (AI) in Scientific Writing

During the final stages of manuscript preparation, the authors used ChatGPT (5.1 and 5.5) to refine sentence structure and improve language clarity. After using this tool, the authors reviewed and edited the content as needed and took full responsibility for the content of the published article. Generative AI tools were not used to simulate, model, or otherwise analyze the data.

## 3 Results

### 3.1 Source Dataset

The data for this study were drawn from a broader project that investigated ambient near‐infrared (NIR) lighting effects on psychophysiological processes (Roddick et al. 2024). Whereas the prior project focused on environmental lighting effects, the present analyses examine how chronic and situational loneliness influence vmHRV reactivity to cognitive demands. Loneliness was not examined as part of the prior project.

Under the broader protocol, participants completed the full cognitive task battery twice—once under each of two counterbalanced lighting conditions (with and without NIR) corresponding to the two sequential blocks described in Section 2.2. The differences between the lighting conditions were designed not to be visually apparent to participants, falling either outside the typical range of human visual detection or at its extreme edge (Griffin et al. 1947; Roberts 2007; Rodieck 1998). During manipulation checks and debriefing, none of the participants mentioned the lighting when asked about the study hypothesis or whether they had noticed anything unusual during the session.

To further verify that ambient lighting did not confound the present results, it was tested as a covariate in all relevant analyses. The inclusion or exclusion of lighting condition did not alter the direction or significance of any associations between loneliness (chronic or situational) and vmHRV reactivity, and there is not a theoretical rationale to expect a relationship between loneliness, a social emotion, and NIR exposure in the ambient environment. As such, all analyses were conducted on data collapsed across lighting conditions.

### 3.2 Analytic Strategy and Model Specification

Multilevel modeling was used to examine the effects of chronic loneliness and situational loneliness on vmHRV reactivity during distinct cognitive challenge tasks. Separate linear mixed‐effects models (LMMs) were specified for each cognitive task to test whether chronic loneliness predicted blunted vmHRV reactivity and whether situational loneliness predicted either blunted or heightened vmHRV reactivity. Each initial model included period (0 = initial rest, 1 = task), loneliness (chronic or situational), and their interaction (loneliness × period). A random intercept for participant was specified in all models to account for baseline differences in resting vmHRV.

Preliminary analyses indicated that block order (i.e., whether the cognitive task was completed during the first or second block) significantly predicted vmHRV reactivity across all cognitive tasks, with consistently greater vmHRV decreases observed during the second block (all *p*s ≤ 0.006). To account for this systematic variance, block order and its interaction with period were retained in all models. Block order and period were modeled as within‐participant factors, whereas loneliness was modeled as a between‐participant predictor. Importantly, the direction and significance of the associations between loneliness and vmHRV reactivity remained stable regardless of whether block order and its interaction were included or not. To further evaluate the robustness of findings across blocks, sensitivity analyses were conducted using only the data from either the first or the second block (see Section 3.7).

Potential covariates—including demographic characteristics, health behaviors, and psychological factors (e.g., depression, anxiety, perceived stress)—were evaluated for their associations with vmHRV reactivity. Variables that showed significant associations with vmHRV reactivity were included as covariates in interim models to test whether loneliness effects remained after adjusting for these factors. Covariates that no longer reached significance once loneliness was accounted for were excluded from the final covariate‐adjusted models for parsimony. All analyses were conducted in R (version 4.4.3; R Core Team 2025).

### 3.3 Power Analysis

As the present analyses were conducted using data from a broader project originally designed to examine ambient lighting effects, an a priori power analysis was not conducted specifically for the loneliness and vmHRV hypotheses. To evaluate statistical power for the present analyses, we conducted an empirical power analysis using Monte Carlo simulation. Simulated datasets were generated to mirror the present study design, including two experimental blocks and repeated vmHRV observations during baseline rest and cognitive task periods, using RMSSD as the primary vmHRV metric. Parameter estimates were based on the initial cognitive task LMMs, with power estimated across a range of loneliness × period interaction effects corresponding to the significant effects observed in the initial chronic loneliness models (*β* = 0.06–0.10). Power was defined as the proportion of 1000 simulated datasets in which the chronic loneliness × period interaction was statistically significant at *α* = 0.05. With *N* = 151, estimated power ranged from approximately 0.68 to 0.97 across this range of interaction effects. Estimated power was approximately 0.79 for *β* = 0.07 and exceeded 0.88 to detect effects of *β* ≥ 0.08.

### 3.4 Chronic Loneliness and vmHRV Reactivity (H1)

Chronic loneliness was significantly associated with vmHRV reactivity during four of the five cognitive challenge tasks: attention, perception, working memory, and executive function. In these four tasks, higher chronic loneliness consistently predicted blunted vmHRV reactivity, reflected by smaller decreases (or increases) in vmHRV from baseline to task (see Table 2 for initial models; see Figures 1, 2, 3, 4). In all cases where chronic loneliness significantly predicted vmHRV reactivity in the initial models, the association remained significant after adjusting for relevant covariates (see Tables 3, 4, 5, 6; see Section 3.8). Overall, chronic loneliness emerged as a predictor of blunted vmHRV reactivity to cognitive challenge, with the largest effect sizes observed for working memory and executive function.

**TABLE 2:** Initial linear mixed models predicting vmHRV reactivity to cognitive tasks from chronic loneliness.

	*β*	SE	*t*	df	95% CI	*p*
Attention						
Period	0.06	0.03	1.60	1650.06	[−0.01, 0.12]	0.111
Block order	0.40	0.03	13.82	1651.46	[0.34, 0.46]	< 0.001***
Chronic loneliness	0.10	0.07	1.38	153.20	[−0.04, 0.24]	0.169
Block order × period	−0.21	0.05	−4.13	1650.15	[−0.30, −0.11]	< 0.001***
Chronic loneliness × period	0.06	0.02	2.59	1650.06	[0.02, 0.11]	0.010**
Perception						
Period	0.25	0.03	8.62	1946.08	[0.19, 0.31]	< 0.001***
Block order	0.40	0.03	14.54	1947.08	[0.34, 0.45]	< 0.001***
Chronic loneliness	0.10	0.07	1.34	153.68	[−0.04, 0.24]	0.184
Block order × period	−0.21	0.04	−5.10	1946.13	[−0.29, −0.13]	< 0.001***
Chronic loneliness × period	0.06	0.02	2.69	1946.06	[0.02, 0.10]	0.007**
Short‐term memory						
Period	0.08	0.03	2.22	1788.63	[0.01, 0.14]	0.026*
Block order	0.42	0.03	13.91	1789.35	[0.36, 0.47]	< 0.001***
Chronic loneliness	0.10	0.07	1.43	154.11	[−0.04, 0.24]	0.154
Block order × period	−0.16	0.05	−3.22	1788.39	[−0.25, −0.06]	0.001**
Chronic loneliness × period	0.03	0.02	1.32	1788.74	[−0.02, 0.08]	0.186
Working memory						
Period	0.02	0.04	0.40	1404.76	[−0.06, 0.10]	0.689
Block order	0.41	0.03	14.68	1405.55	[0.36, 0.47]	< 0.001***
Chronic loneliness	0.10	0.07	1.40	151.61	[−0.04, 0.24]	0.164
Block order × period	−0.16	0.06	−2.74	1404.38	[−0.27, −0.05]	0.006**
Chronic loneliness × period	0.10	0.03	3.52	1404.97	[0.05, 0.16]	< 0.001***
Executive function						
Period	−0.08	0.04	−2.28	1562.78	[−0.15, −0.01]	0.023*
Block order	0.41	0.03	14.62	1563.66	[0.36, 0.47]	< 0.001***
Chronic loneliness	0.10	0.07	1.42	152.70	[−0.04, 0.24]	0.157
Block order × period	−0.27	0.05	−5.18	1562.50	[−0.37, −0.17]	< 0.001***
Chronic loneliness × period	0.07	0.03	2.69	1563.11	[0.02, 0.12]	0.007**

*Note:* VmHRV is quantified using the root mean square of successive differences (RMSSD). Period is coded 0 = baseline, 1 = task. Block order is coded 0 = first block, 1 = second block.

Abbreviations: CI, confidence interval; SE, standard error; vmHRV, vagally‐mediated heart rate variability.

*p < 0.05, **p < 0.01, ***p < 0.001.

**FIGURE 1:**
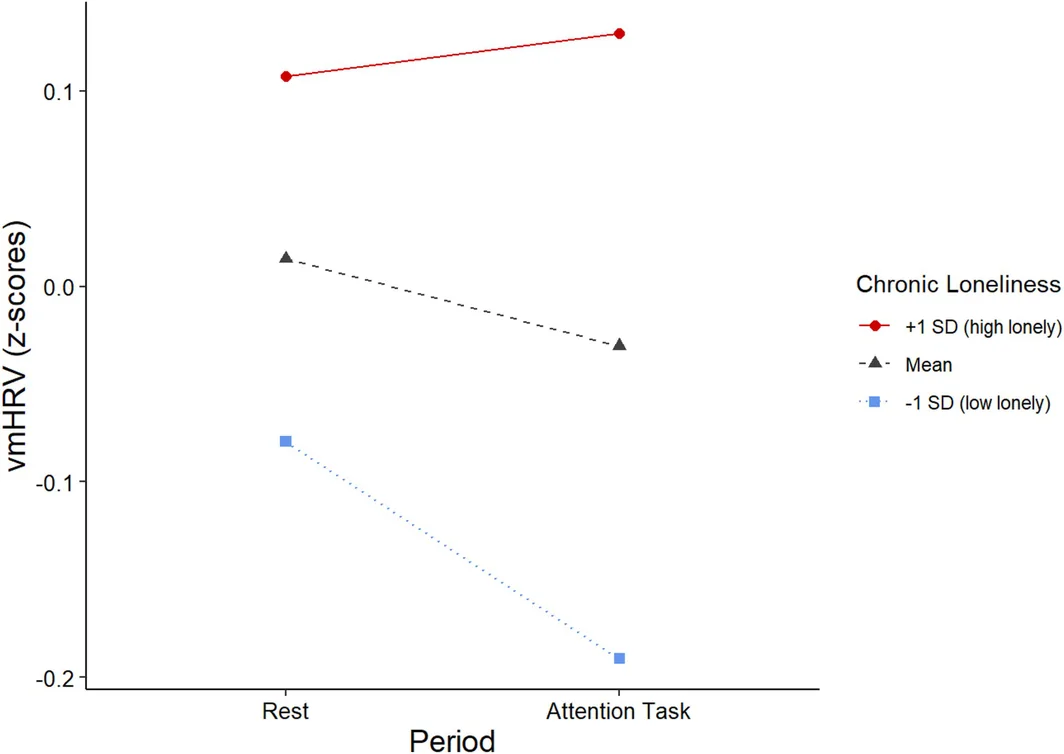
Predicted vagally mediated heart rate variability (vmHRV) reactivity to the attention task by levels of chronic loneliness. VmHRV is quantified as the root mean square of successive differences (RMSSD), expressed in standardized units (*z*‐scores). Lines depict estimated vmHRV trajectories from linear mixed‐effects models at −1 SD, mean, and +1 SD of chronic loneliness (UCLA Loneliness Scale modified for a 5‐year timeframe; Russell 1996) to illustrate the significant association between higher chronic loneliness and blunted cardiac vagal withdrawal.

**TABLE 3:** Covariate‐adjusted LMM predicting vmHRV reactivity to the attention task from chronic loneliness.

	*β*	SE	*t*	df	95% CI	*p*
Period	0.21	0.06	3.70	1635.27	[0.10, 0.33]	< 0.001***
Block order	0.40	0.03	13.86	1636.49	[0.34, 0.46]	< 0.001***
Chronic loneliness	0.10	0.07	1.41	147.24	[−0.04, 0.23]	0.160
Sex	0.45	0.17	2.65	147.13	[0.12, 0.78]	0.009**
Body mass index (BMI)	−0.01	0.07	−0.09	146.88	[−0.15, 0.13]	0.926
Hours slept	−0.03	0.07	−0.42	147.34	[−0.16, 0.10]	0.679
Average caffeine intake	0.19	0.07	2.60	143.00	[0.05, 0.34]	0.010*
Habitual cognitive reappraisal	0.06	0.07	0.91	147.43	[−0.07, 0.20]	0.364
Block order × period	−0.21	0.05	−4.19	1635.14	[−0.31, −0.11]	< 0.001***
Sex × period	−0.21	0.06	−3.47	1635.40	[−0.32, −0.09]	< 0.001***
BMI × period	−0.06	0.03	−2.53	1635.08	[−0.11, −0.01]	0.011*
Hours slept × period	0.08	0.02	3.29	1635.22	[0.03, 0.13]	0.001**
Cognitive reappraisal × period	−0.05	0.03	−2.18	1635.32	[−0.10, −0.01]	0.030*
Chronic loneliness × period	0.06	0.03	2.36	1635.05	[0.01, 0.11]	0.018*

*Note:* VmHRV is quantified using the root mean square of successive differences (RMSSD). Period is coded 0 = baseline, 1 = task. Block order is coded 0 = first block, 1 = second block. Sex is coded 0 = male, 1 = female.

Abbreviations: CI, confidence interval; LMM, linear mixed model; SE, standard error; vmHRV, vagally‐mediated heart rate variability.

*p < 0.05, **p < 0.01, ***p < 0.001.

For the attention task (Figure 1), chronic loneliness significantly predicted blunted vmHRV reactivity, as indicated by a significant loneliness × period interaction in the initial model (*β* = 0.06, SE = 0.02, *t*(1650) = 2.59, *p* = 0.010, 95% CI [0.02, 0.11]; Table 2). Likewise, in the final covariate‐adjusted model, chronic loneliness remained a significant predictor of blunted vmHRV withdrawal during the attention task (*β* = 0.06, SE = 0.03, *t*(1635) = 2.36, *p* = 0.018, 95% CI [0.01, 0.11]; Table 3).

Similarly, for the perception task (Figure 2), chronic loneliness significantly predicted blunted vmHRV reactivity in the initial model (*β* = 0.06, SE = 0.02, *t*(1946) = 2.69, *p* = 0.007, 95% CI [0.02, 0.10]; Table 2). This effect remained significant, though slightly attenuated, in the final covariate‐adjusted model (*β* = 0.04, SE = 0.02, *t*(1944) = 2.03, *p* = 0.042, 95% CI [0.00, 0.08]; Table 4).

**FIGURE 2:**
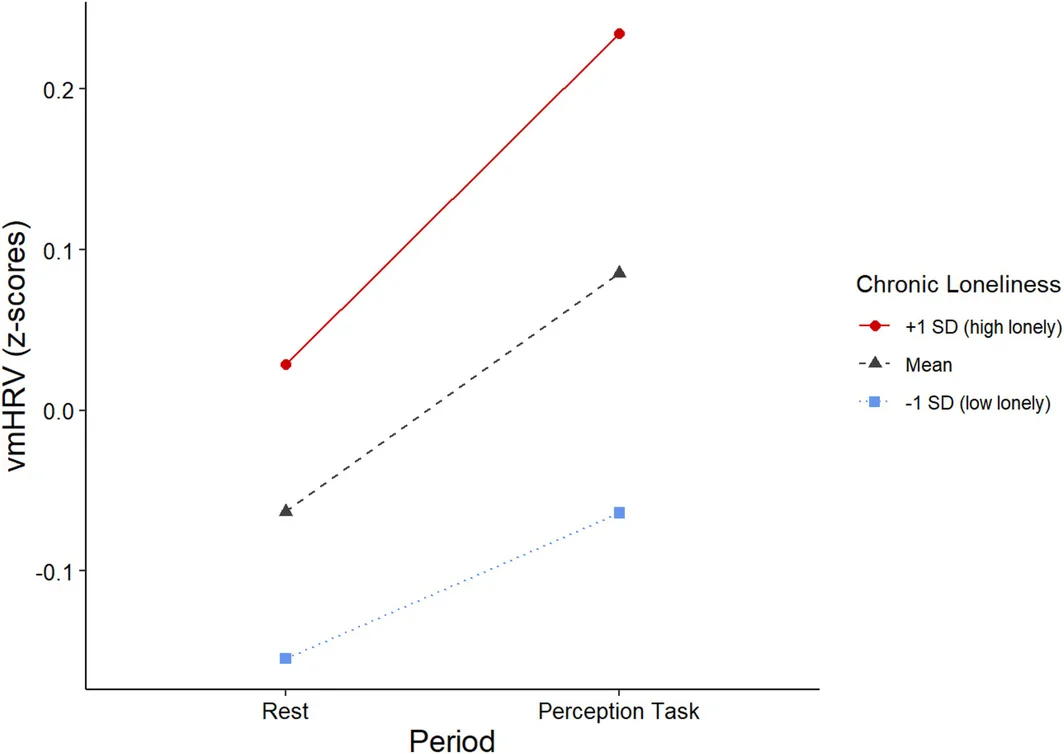
Predicted vagally mediated heart rate variability (vmHRV) reactivity to the perception task by levels of chronic loneliness. VmHRV is quantified as the root mean square of successive differences (RMSSD), expressed in standardized units (*z*‐scores). Lines depict estimated vmHRV trajectories from linear mixed‐effects models at −1 SD, mean, and +1 SD of chronic loneliness (UCLA Loneliness Scale modified for a 5‐year timeframe; Russell 1996) to illustrate the significant association between higher chronic loneliness and blunted cardiac vagal withdrawal.

**TABLE 4:** Covariate‐adjusted LMM predicting vmHRV reactivity to the perception task from chronic loneliness.

	*β*	SE	*t*	df	95% CI	*p*
Period	0.25	0.03	8.61	1944.08	[0.19, 0.31]	< 0.001***
Block order	0.40	0.03	14.58	1945.14	[0.34, 0.45]	< 0.001***
Chronic loneliness	0.09	0.07	1.29	149.88	[−0.04, 0.22]	0.201
Sex	0.41	0.17	2.46	145.04	[0.09, 0.74]	0.015*
Hours slept	−0.02	0.07	−0.30	149.97	[−0.15, 0.11]	0.762
Average caffeine intake	0.19	0.07	2.67	145.02	[0.05, 0.33]	0.008**
Habitual cognitive reappraisal	0.06	0.07	0.83	150.05	[−0.08, 0.19]	0.410
Block order × period	−0.21	0.04	−5.05	1944.14	[−0.29, −0.13]	< 0.001***
Hours slept × period	0.05	0.02	2.20	1944.20	[0.00, 0.09]	0.028*
Cognitive reappraisal × period	−0.09	0.02	−4.44	1944.28	[−0.13, −0.05]	< 0.001***
Chronic loneliness × period	0.04	0.02	2.03	1944.04	[0.00, 0.08]	0.042*

*Note:* VmHRV is quantified using the root mean square of successive differences (RMSSD). Period is coded 0 = baseline, 1 = task. Block order is coded 0 = first block, 1 = second block. Sex is coded 0 = male, 1 = female.

Abbreviations: CI, confidence interval; LMM, linear mixed model; SE, standard error; vmHRV, vagally‐mediated heart rate variability.

*p < 0.05, **p < 0.01, ***p < 0.001.

Chronic loneliness was a robust predictor of blunted vmHRV reactivity during the working memory task (Figure 3) in both the initial model (*β* = 0.10, SE = 0.03, *t*(1405) = 3.52, *p* < 0.001, 95% CI [0.05, 0.16]; Table 2) and in the final covariate‐adjusted model (*β* = 0.10, SE = 0.03, *t*(1404) = 3.47, *p* < 0.001, 95% CI [0.04, 0.16]; Table 5). Notably, although depressive symptoms and perceived stress were initially associated with blunted vmHRV reactivity to working memory demands in preliminary analyses, both depressive symptoms and perceived stress became non‐significant after adjusting for chronic loneliness and were excluded from the final model.

**FIGURE 3:**
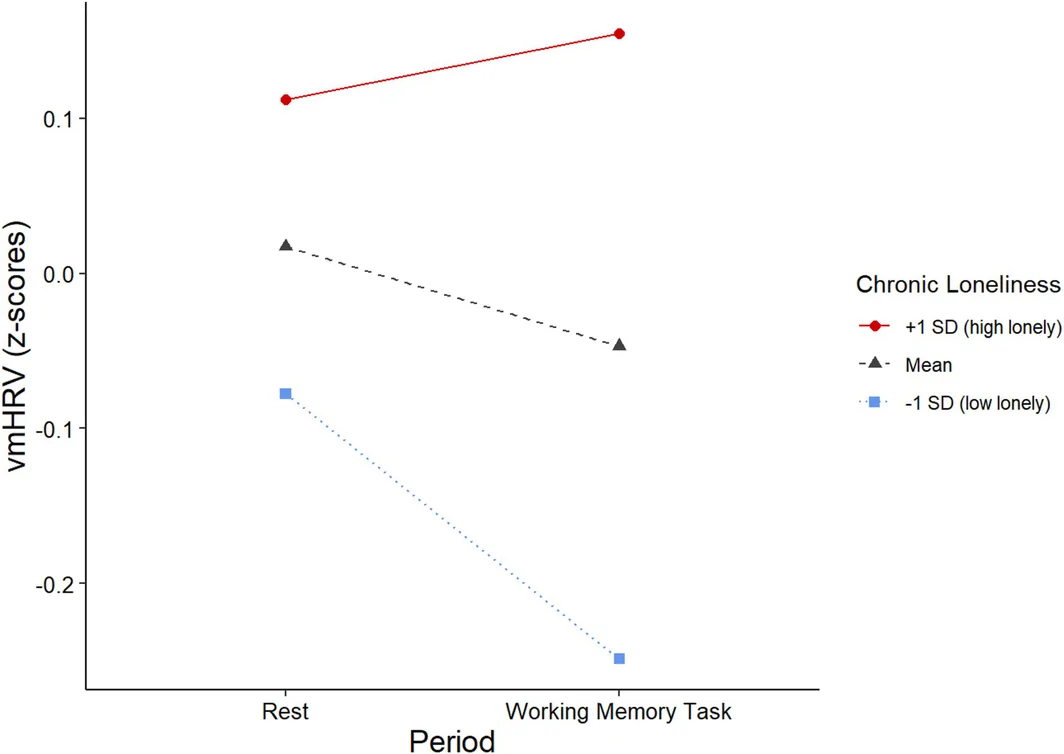
Predicted vagally mediated heart rate variability (vmHRV) reactivity to the working memory task by levels of chronic loneliness. VmHRV is quantified as the root mean square of successive differences (RMSSD), expressed in standardized units (*z*‐scores). Lines depict estimated vmHRV trajectories from linear mixed‐effects models at −1 SD, mean, and +1 SD of chronic loneliness (UCLA Loneliness Scale modified for a 5‐year timeframe; Russell 1996) to illustrate the significant association between higher chronic loneliness and blunted cardiac vagal withdrawal.

**TABLE 5:** Covariate‐adjusted LMM predicting vmHRV reactivity to the working memory task from chronic loneliness.

	*β*	SE	*t*	df	95% CI	*p*
Period	0.02	0.04	0.44	1403.80	[−0.06, 0.10]	0.663
Block order	0.41	0.03	14.71	1404.67	[0.36, 0.47]	< 0.001***
Chronic loneliness	0.09	0.07	1.26	148.77	[−0.05, 0.22]	0.208
Sex	0.43	0.17	2.56	146.18	[0.10, 0.75]	0.011*
Hours slept	−0.02	0.07	−0.35	148.87	[−0.16, 0.11]	0.724
Average caffeine intake	0.17	0.07	2.44	146.04	[0.04, 0.31]	0.016*
Block order × period	−0.16	0.06	−2.76	1403.40	[−0.28, −0.05]	0.006**
Hours slept × period	0.07	0.03	2.53	1404.31	[0.02, 0.13]	0.012*
Chronic loneliness × period	0.10	0.03	3.47	1404.03	[0.04, 0.16]	< 0.001***

*Note:* VmHRV is quantified using the root mean square of successive differences (RMSSD). Period is coded 0 = baseline, 1 = task. Block order is coded 0 = first block, 1 = second block. Sex is coded 0 = male, 1 = female.

Abbreviations: CI, confidence interval; LMM, linear mixed model; SE, standard error; vmHRV, vagally‐mediated heart rate variability.

*p < 0.05, **p < 0.01, ***p < 0.001.

Chronic loneliness also significantly predicted blunted vmHRV reactivity during the executive function task (*β* = 0.07, SE = 0.03, *t*(1563) = 2.69, *p* = 0.007, 95% CI [0.02, 0.12]; Table 2; see Figure 4). This association remained significant in the final covariate‐adjusted model (*β* = 0.07, SE = 0.03, *t*(1550) = 2.89, *p* = 0.004, 95% CI [0.02, 0.12]; Table 6).

**FIGURE 4:**
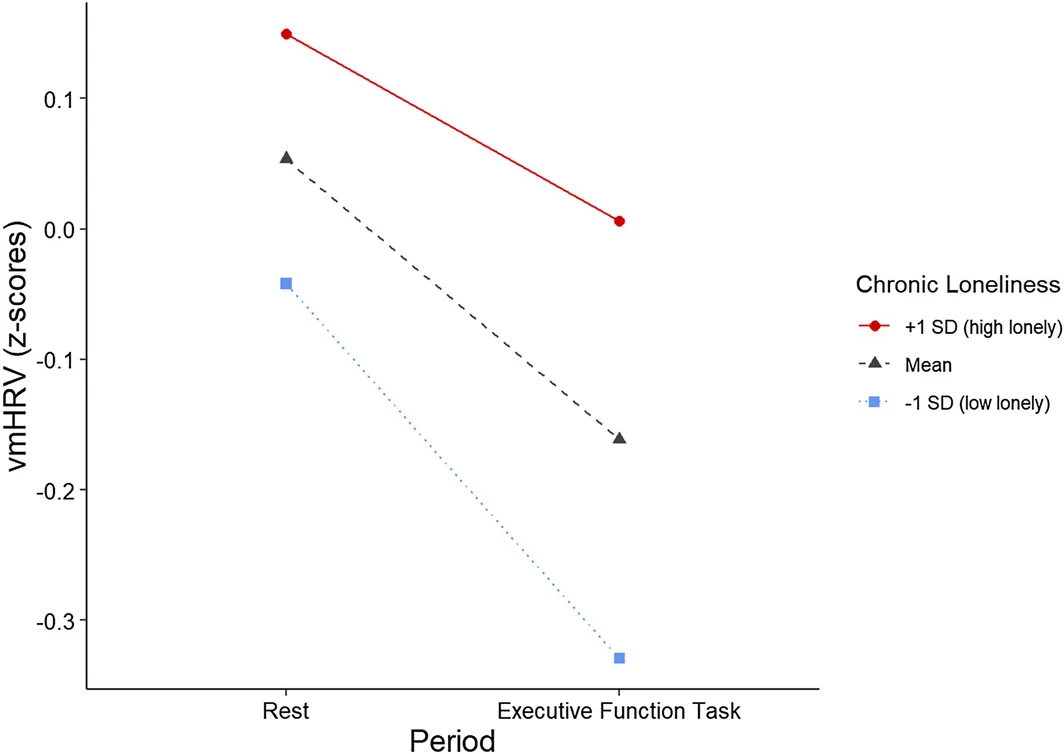
Predicted vagally mediated heart rate variability (vmHRV) reactivity to the executive function task by levels of chronic loneliness. VmHRV is quantified as the root mean square of successive differences (RMSSD), expressed in standardized units (*z*‐scores). Lines depict estimated vmHRV trajectories from linear mixed‐effects models at −1 SD, mean, and +1 SD of chronic loneliness (UCLA Loneliness Scale modified for a 5‐year timeframe; Russell 1996) to illustrate the significant association between higher chronic loneliness and blunted cardiac vagal withdrawal.

**TABLE 6:** Covariate‐adjusted LMM predicting vmHRV reactivity to the executive function task from chronic loneliness.

	*β*	SE	*t*	df	95% CI	*p*
Period	0.08	0.06	1.22	1551.00	[−0.05, 0.20]	0.224
Block order	0.42	0.03	14.63	1551.00	[0.36, 0.47]	< 0.001***
Chronic loneliness	0.09	0.07	1.34	148.90	[−0.04, 0.22]	0.184
Sex	0.46	0.17	2.74	148.80	[0.13, 0.79]	0.007**
Body mass index (BMI)	0.00	0.07	0.04	148.50	[−0.13, 0.14]	0.966
Average caffeine intake	0.18	0.07	2.46	145.00	[0.04, 0.32]	0.015*
Block order × period	−0.27	0.05	−5.24	1550.00	[−0.37, −0.17]	< 0.001***
Sex × period	−0.19	0.06	−3.01	1551.00	[−0.32, −0.07]	0.003**
BMI × period	−0.08	0.03	−3.06	1550.00	[−0.13, −0.03]	0.002**
Chronic loneliness × period	0.07	0.03	2.89	1550.00	[0.02, 0.12]	0.004**

*Note:* VmHRV is quantified using the root mean square of successive differences (RMSSD). Period is coded 0 = baseline, 1 = task. Block order is coded 0 = first block, 1 = second block. Sex is coded 0 = male, 1 = female.

Abbreviations: CI, confidence interval; LMM, linear mixed model; SE, standard error; vmHRV, vagally‐mediated heart rate variability.

*p < 0.05, **p < 0.01, ***p < 0.001.

Chronic loneliness did not significantly predict vmHRV reactivity during the short‐term memory task (*β* = 0.03, SE = 0.02, *t*(1789) = 1.32, *p* = 0.186, 95% CI [−0.02, 0.08]; Table 2). As such, no covariate‐adjusted model was conducted for this task.

### 3.5 Situational Loneliness and vmHRV Reactivity (H2)

Compared to chronic loneliness, situational loneliness was not as consistently associated with vmHRV reactivity across cognitive tasks. However, when significant or marginal associations were observed, the direction of the effect indicated that situational loneliness (similar to chronic loneliness) predicted blunted vmHRV reactivity during cognitive challenge.

Situational loneliness significantly predicted blunted vmHRV reactivity during the perception task, as indicated by a significant loneliness × period interaction in both the initial model (*β* = 0.05, SE = 0.02, *t*(1946) = 2.59, *p* = 0.010, 95% CI [0.01, 0.09]) and the final covariate‐adjusted model (*β* = 0.04, SE = 0.02, *t*(1944) = 2.05, *p* = 0.041, 95% CI [0.00, 0.08]; Tables S2 and S3; see Section 3.8).

For the short‐term memory task, situational loneliness significantly predicted blunted vmHRV reactivity in the initial model (*β* = 0.05, SE = 0.02, *t*(1789) = 2.18, *p* = 0.029, 95% CI [0.01, 0.10]; Table S2). However, this effect did not remain significant after adjusting for covariates, including perceived stress (Table S4; see Section 3.8).

For the working memory task, situational loneliness was marginally associated with blunted vmHRV reactivity in the initial model (*β* = 0.06, SE = 0.03, *t*(1405) = 1.92, *p* = 0.055, 95% CI [−0.00, 0.12]; Table S2), but this association was not significant after adjusting for covariates (Table S5). Similarly, the association between situational loneliness and vmHRV reactivity became non‐significant when chronic loneliness was accounted for (see Section 3.6).

Situational loneliness was not significantly associated with vmHRV reactivity during either the attention task (*β* = 0.01, SE = 0.02, *t*(1650) = 0.46, *p* = 0.646, 95% CI [−0.04, 0.06]) or the executive function task (*β* = 0.03, SE = 0.03, *t*(1563) = 0.99, *p* = 0.322, 95% CI [−0.03, 0.08]; Table S2).

### 3.6 Chronic Loneliness × Situational Loneliness Interactions

Exploratory analyses were conducted to examine whether chronic and situational loneliness interacted in predicting vmHRV reactivity across cognitive tasks. For each task, linear mixed‐effects models were specified that included period, block order, chronic loneliness, situational loneliness, and all relevant two‐way and three‐way interactions (see Tables 7 and 8 in the main text and Tables S6–S9 for full model outputs). These analyses were further adjusted for covariates only when the initial model indicated a significant interaction of chronic loneliness, situational loneliness, and period.

**TABLE 7:** Initial LMM predicting vmHRV reactivity to the working memory task from chronic and situational loneliness.

	*β*	SE	*t*	df	95% CI	*p*
Period	−0.01	0.04	−0.29	1402.78	[−0.09, 0.07]	0.772
Block order	0.41	0.03	14.68	1403.52	[0.36, 0.47]	< 0.001***
Chronic loneliness	0.10	0.08	1.35	149.60	[−0.05, 0.26]	0.178
Situational loneliness	−0.03	0.08	−0.40	149.74	[−0.18, 0.12]	0.687
Block order × period	−0.16	0.06	−2.69	1402.37	[−0.27, −0.04]	0.007**
Chronic loneliness × period	0.10	0.03	3.14	1403.08	[0.04, 0.16]	0.002**
Situational loneliness × period	0.02	0.03	0.72	1403.06	[−0.04, 0.09]	0.474
Chronic loneliness × situational loneliness	−0.07	0.08	−0.91	149.61	[−0.21, 0.08]	0.363
Chronic × situational × period	0.07	0.03	2.31	1402.83	[0.01, 0.13]	0.021*

*Note:* VmHRV is quantified using the root mean square of successive differences (RMSSD). Period is coded 0 = baseline, 1 = task. Block order is coded 0 = first block, 1 = second block.

Abbreviations: CI, confidence interval; LMM, linear mixed model; SE, standard error; vmHRV, vagally‐mediated heart rate variability.

*p < 0.05, **p < 0.01, ***p < 0.001.

**TABLE 8:** Covariate‐adjusted LMM predicting vmHRV reactivity to the working memory task from chronic and situational loneliness.

	*β*	SE	*t*	df	95% CI	*p*
Period	−0.01	0.04	−0.21	1401.82	[−0.09, 0.07]	0.832
Block order	0.41	0.03	14.70	1402.63	[0.36, 0.47]	< 0.001***
Chronic loneliness	0.08	0.07	1.10	146.76	[−0.06, 0.23]	0.272
Situational loneliness	−0.01	0.07	−0.09	146.94	[−0.15, 0.14]	0.929
Sex	0.42	0.17	2.47	144.20	[0.09, 0.74]	0.015*
Average caffeine intake	0.18	0.07	2.48	144.04	[0.04, 0.31]	0.014*
Hours slept	−0.02	0.07	−0.30	146.80	[−0.15, 0.11]	0.768
Hours slept × period	0.07	0.03	2.44	1402.28	[0.01, 0.13]	0.015*
Block order × period	−0.16	0.06	−2.71	1401.38	[−0.27, −0.04]	0.007**
Chronic loneliness × period	0.10	0.03	3.01	1402.18	[0.03, 0.16]	0.003**
Situational loneliness × period	0.03	0.03	0.91	1402.14	[−0.03, 0.09]	0.362
Chronic loneliness × situational loneliness	−0.07	0.07	−0.93	146.73	[−0.21, 0.07]	0.352
Chronic × situational × period	0.07	0.03	2.11	1401.84	[0.00, 0.13]	0.035*

*Note:* VmHRV is quantified using the root mean square of successive differences (RMSSD). Period is coded 0 = baseline, 1 = task. Block order is coded 0 = first block, 1 = second block. Sex is coded 0 = male, 1 = female.

Abbreviations: CI, confidence interval; LMM, linear mixed model; SE, standard error; vmHRV, vagally‐mediated heart rate variability.

*p < 0.05, **p < 0.01, ***p < 0.001.

A significant three‐way interaction between chronic loneliness, situational loneliness, and period emerged for the working memory task, whereas marginal interactions were observed for the executive function task and the short‐term memory task. No significant three‐way interactions were found for the attention or perception tasks. In models where the three‐way interaction was significant or marginal, the positive coefficient indicated an additive effect—vmHRV withdrawal during cognitive challenge was especially blunted when both chronic and situational loneliness were elevated.

Specifically, for the working memory task, the chronic loneliness × situational loneliness × period interaction was significant in the initial model (*β* = 0.07, SE = 0.03, *t*(1403) = 2.31, *p* = 0.021, 95% CI [0.01, 0.13]; Table 7), and remained significant after adjusting for covariates (*β* = 0.07, SE = 0.03, *t*(1402) = 2.11, *p* = 0.035, 95% CI [0.00, 0.13]; Table 8). This exploratory finding suggests that situational loneliness may further amplify the blunting effect of chronic loneliness on vmHRV reactivity during working memory demands. Notably, across both the initial and covariate‐adjusted models, chronic loneliness remained a robust predictor of blunted vmHRV reactivity, whereas situational loneliness did not predict vmHRV reactivity after accounting for chronic loneliness (see Tables 7 and 8).

The three‐way interaction between chronic loneliness, situational loneliness, and period was marginally significant for the executive function task (*β* = 0.05, SE = 0.03, *t*(1561) = 1.96, *p* = 0.051, 95% CI [−0.00, 0.10]; Table S6), as well as for the short‐term memory task (*β* = 0.04, SE = 0.03, *t*(1787) = 1.67, *p* = 0.095, 95% CI [−0.01, 0.09]; Table S7), suggesting similar additive trends, though these findings did not meet the traditional cut‐off for statistical significance and should be interpreted with caution. The three‐way interaction was not significant for the attention task (*β* = 0.02, SE = 0.03, *t*(1648) = 0.72, *p* = 0.473, 95% CI [−0.03, 0.07]; Table S8) or the perception task (*β* = −0.01, SE = 0.02, *t*(1944) = −0.58, *p* = 0.564, 95% CI [−0.06, 0.03]; Table S9).

Across all interaction models, the previously reported associations between chronic loneliness and blunted vmHRV withdrawal remained generally robust. In contrast, associations involving situational loneliness tended to weaken or become non‐significant when chronic loneliness was included in the same model.

### 3.7 Sensitivity Analyses

To evaluate the robustness of the main findings, models testing chronic and situational loneliness as predictors of vmHRV reactivity were re‐run using only data from the first block or only from the second block (see Tables S10–S13). Because each block represents approximately half the dataset, statistical power was reduced, and some effects were attenuated; however, the overall pattern of results was broadly consistent with the main analyses.

For chronic loneliness, the direction and significance of associations observed for each individual block generally aligned with the primary results (see Tables S10 and S11 and Figures S2–S5). Specifically, chronic loneliness significantly or marginally predicted blunted vmHRV reactivity to the attention, working memory, and executive function tasks in both individual blocks. Chronic loneliness also predicted blunted vmHRV reactivity to perception in the first block, though not in the second. Consistent with the main findings, chronic loneliness was not associated with vmHRV reactivity to the short‐term memory task in either block.

For situational loneliness, results of analyses conducted on the individual blocks also mirrored the primary models (see Tables S12 and S13). Specifically, situational loneliness significantly or marginally predicted blunted vmHRV reactivity to the perception task in both blocks and to the short‐term memory task in the second block. The association between situational loneliness and blunted vmHRV reactivity to the working memory task was non‐significant in the first block and marginal in the second block. As in the main analyses, situational loneliness was not significantly associated with vmHRV reactivity to attention or executive function demands in either block.

Patterns of overall vmHRV reactivity (i.e., change from baseline to task across all participants) suggested differences between blocks (Tables S10–S13). During the first block, participants tended to exhibit increases or smaller decreases in vmHRV from baseline, potentially reflecting initial task orientation or the novelty of implementing new instructions. In the second block, vmHRV decreases were more consistent, possibly indicating greater familiarity with the task and more focused cognitive engagement, as the effort involved in processing new instructions was reduced. Across both blocks, the most pronounced vmHRV decreases were observed during the executive function task.

Additional sensitivity analyses were conducted with participants stratified by sex to examine whether patterns of loneliness‐related vmHRV reactivity were similar for females and males (see Tables S14–S17). These analyses should be interpreted cautiously given the smaller male subsample (*n* = 34 males; *n* = 117 females). The female‐only models generally mirrored the full‐sample findings for chronic and situational loneliness, whereas the male‐only models showed less consistent statistical significance but generally similar directions of effects.

Collectively, these sensitivity analyses provide additional support for the robustness of the main findings—particularly the association between chronic loneliness and blunted vmHRV reactivity—across separate subsets of the data.^5^

### 3.8 Covariate Summary

In addition to loneliness, several covariates showed associations with vmHRV reactivity to cognitive demands. Across tasks, block order consistently predicted vmHRV withdrawal (with greater withdrawal occurring in the second block), possibly reflecting increased task familiarity and more focused cognitive engagement, as participants were no longer processing the instructions for the first time. Other covariates showed less consistent associations with vmHRV reactivity. Less self‐reported sleep the previous night, higher BMI, female sex, and greater habitual cognitive reappraisal were significantly associated with greater vmHRV withdrawal during some tasks (after accounting for chronic or situational loneliness; see Tables 3, 4, 5, 6 in the main text and Tables S3–S5). These associations were generally in the same direction as those reported in previous studies (Ferreira et al. 2022; Gerardo et al. 2019; Jentsch and Wolf 2020; Koenig and Thayer 2016; Mezick et al. 2014; Williams et al. 2017). However, these associations were not consistently observed across all the cognitive challenge tasks and did not explain the primary effects of chronic loneliness on vmHRV reactivity. Notably, some variables (e.g., depressive symptoms, perceived stress, average caffeine consumption) no longer predicted vmHRV reactivity once chronic loneliness was included in the same model.

Other variables—including cognitive task performance,^6^ social network size and diversity, anxiety symptoms, expressive suppression, self‐reported typical exercise and sleep habits, alcohol intake, and age—were not significantly associated with vmHRV reactivity during any task. Overall, these results suggest that the effects of chronic loneliness on vmHRV reactivity were not accounted for by other measured covariates, including a wide range of demographic, psychological, and health‐related factors.

## 4 Discussion

The present study examined how temporal dimensions of loneliness—specifically, chronic loneliness experienced over the past 5 years and situational loneliness experienced over the past 24 h—predicted vagally mediated heart rate variability (vmHRV) reactivity across five distinct cognitive challenge tasks. This investigation extends prior work by assessing whether loneliness‐related alterations in neurovisceral regulation generalize beyond visuospatial attention demands (Muhtadie et al. 2015; Roddick and Chen 2021) to encompass other cognitive processes, including attention, perception, short‐term memory, working memory, and executive function.

Consistent with hypotheses, chronic loneliness emerged as a robust predictor of blunted vmHRV reactivity across multiple cognitive challenge tasks. Individuals reporting higher chronic loneliness exhibited smaller decreases—or in some cases, increases—in vmHRV from baseline to task, indicating reduced task‐elicited cardiac vagal withdrawal. This pattern was evident across attention, perception, working memory, and executive function tasks, with the largest effects observed for working memory and executive function, suggesting these processes may be particularly sensitive to loneliness‐related disruptions in cognitive‐autonomic integration.

In contrast, situational loneliness demonstrated weaker and less consistent associations with vmHRV reactivity. However, when significant or marginal effects were observed, they likewise pointed toward blunted vmHRV reactivity, rather than the greater physiological mobilization that could be considered an extension of theoretical perspectives on the potential adaptive benefits of transient loneliness (Cacioppo et al. 2014). In exploratory analyses, the combination of high chronic and high situational loneliness was associated with especially blunted vmHRV reactivity during working memory demands, and marginally so for executive function demands, suggesting a potential additive burden when both forms of loneliness co‐occur.

Importantly, the associations between chronic loneliness and blunted vmHRV reactivity proved robust across multiple analytic approaches. The effects of chronic loneliness remained significant after adjusting for an extensive set of potential confounding influences—including depressive symptoms, perceived stress, anxiety, habitual emotion regulation strategies (reappraisal and suppression), social network size and diversity, sleep duration, exercise and sleep habits, caffeine and alcohol use, biological sex, and body mass index. The findings also remained stable when controlling for block order effects, were generally consistent across sensitivity analyses restricted to each task block separately, and were directionally corroborated using an alternative frequency‐domain index of vmHRV (HF‐HRV, ln ms^2^). Although the effect sizes were small, this magnitude aligns with expectations for complex physiological systems shaped by multiple biological, psychological, and environmental influences (Laborde et al. 2017; Sammito et al. 2024). Even modest, reliable effects may have health relevance, as persistent physiological differences can accumulate over time to influence disease risk (Funder and Ozer 2019). Collectively, these results underscore the strength of chronic loneliness as a predictor of blunted cardiac vagal reactivity during cognitive demands, highlighting a potentially pervasive impact on neurovisceral regulation that spans multiple cognitive processes.

The present findings suggest a distinctive role of chronic loneliness in autonomic‐cognitive functioning. The effects of chronic loneliness persisted even after accounting for numerous psychological, behavioral, and demographic covariates, indicating that chronic loneliness may represent an independent correlate of altered neurovisceral regulation—beyond what might be explained by general psychological distress, maladaptive emotion regulation, health‐related behaviors, or other lifestyle factors.

This interpretation is reinforced by the observation that habitual cognitive reappraisal—a generally adaptive emotion regulation strategy—was associated with greater vmHRV withdrawal during some tasks, indicative of more flexible cardiac vagal control. This stands in contrast to the blunted vmHRV reactivity linked to chronic loneliness, pointing to potentially distinct pathways through which loneliness and emotion regulation may influence neurovisceral integration. Notably, the effects of chronic loneliness on vmHRV reactivity remained significant even after adjusting for both habitual cognitive reappraisal and expressive suppression, supporting the possibility that loneliness exerts a unique influence on cardiac vagal activity not reducible to differences in emotion regulation or other measured factors.

### 4.1 Chronic Versus Situational Loneliness: Theoretical Implications

The present findings contribute to a broader understanding of how loneliness may influence physiological regulatory processes that integrate cognitive, affective, and autonomic functioning. In particular, the findings align closely with the neurovisceral integration model (Thayer and Lane 2009), which proposes that adaptive regulation of cognitive and autonomic responses relies on the coordinated activity of prefrontal and subcortical networks. Although the neurovisceral integration model does not explicitly conceptualize loneliness as a driver of such processes, applying its principles here suggests that chronic loneliness may disrupt neural circuits responsible for dynamically modulating cardiac vagal activity. This interpretation is reinforced by conceptual accounts positing that loneliness affects engagement of prefrontal‐limbic circuits involved in cognitive and affective regulation (Cacioppo and Hawkley 2009; Quadt et al. 2020), as well as by broader evidence that blunted cardiovascular and cortisol reactivity to stress reflects suboptimal functioning of fronto‐limbic systems critical for motivation and adaptive engagement (Carroll et al. 2017). Thus, the observed blunted vmHRV reactivity among individuals reporting greater chronic loneliness may indicate reduced top‐down modulation of physiological states by prefrontal systems in the face of cognitive demand.

These findings also align with the concept of social allostatic load (Quadt et al. 2020), wherein chronic loneliness is thought to embed chronic dysregulation across neural and autonomic systems. That chronic loneliness predicted blunted cardiac vagal withdrawal across a range of cognitive challenge tasks—including those placing demands on attention, perception, working memory, and executive function—supports the notion of a generalized disruption in cognitive‐autonomic integration spanning multiple cognitive processes. Such pervasive blunting of task‐elicited vmHRV reactivity may represent an early stage of the physiological embedding of chronic loneliness, preceding the emergence of more overt health consequences.

Notably, the strongest associations emerged during the working memory and executive function tasks, which place particularly high demands on prefrontal cortical systems implicated in maintaining and manipulating information and resolving interference from conflicting stimuli (Aben et al. 2012; Cowan 2008; Diamond 2013; Friedman and Robbins 2022). Conversely, chronic loneliness did not significantly predict vmHRV reactivity during the short‐term memory task. One possibility is that the short‐term memory task may place fewer demands on the regulatory circuits integrating cognitive and autonomic activity (Aben et al. 2012; Cowan 2008), and may consequently have been less sensitive to detecting loneliness‐related disruptions in neurovisceral regulation. Similarly, the initial association between situational loneliness and blunted vmHRV reactivity during the short‐term memory task became non‐significant after covariate adjustment, further suggesting that findings for this task should be interpreted cautiously. This attenuation may reflect the lower regulatory demands of the short‐term memory task, as well as potential conceptual and temporal overlap between situational loneliness and other proximal psychological factors, such as perceived stress.

### 4.2 Chronic Versus Situational Loneliness: Distinct and Additive Associations With vmHRV Reactivity

The differing strength of associations for chronic versus situational loneliness was evident not only in their links to vmHRV reactivity but also in their broader psychological correlates. Chronic and situational loneliness were moderately correlated with each other, indicating that while related, they capture distinct facets of the loneliness experience. Both forms of loneliness were positively correlated with depressive symptoms, perceived stress, anxiety, and habitual expressive suppression, and marginally negatively correlated with habitual cognitive reappraisal. However, these associations were generally stronger for situational loneliness, suggesting that day‐level loneliness may more closely track immediate mood and stress states, whereas chronic loneliness reflects a more stable, trait‐like vulnerability that may be more deeply embedded in neurophysiological regulation.

Although both chronic and situational loneliness were examined as predictors of vmHRV reactivity, the present findings underscore the role of chronic loneliness in shaping patterns of cardiac vagal regulation. Situational loneliness generally showed weaker and less consistent associations with vmHRV reactivity, and its effects sometimes diminished when chronic loneliness was included in the same models—pointing to chronic loneliness as the dominant driver of dysregulated cardiac vagal responses to cognitive demand. Nonetheless, when significant or marginal effects of situational loneliness did emerge, they likewise indicated blunted vmHRV reactivity, echoing the pattern observed for chronic loneliness rather than heightened physiological mobilization in the form of exaggerated vmHRV reactivity. This may suggest that situational feelings of loneliness do not upregulate physiological readiness for cognitive and social demands, but instead may exert an amplifying effect when chronic loneliness is also present.

Indeed, exploratory analyses pointed to a possible additive burden when both forms of loneliness were elevated. Participants reporting high levels of both chronic and situational loneliness exhibited especially blunted vmHRV reactivity during the working memory task, with a similar though marginal pattern observed for the executive function task. These findings tentatively suggest that situational loneliness may exacerbate the physiological impact of chronic loneliness, amplifying disruptions in cardiac vagal regulation during cognitively demanding contexts.

### 4.3 Limitations and Future Directions

Several limitations of the present study should be acknowledged, alongside important considerations for future research. The sample was predominantly female, which limits the extent to which the present findings can be generalized to males. Sensitivity analyses suggested that the female‐only models broadly reproduced the full‐sample findings for associations between loneliness and vmHRV reactivity, whereas the male‐only models were less consistently significant but generally similar in direction. Overall, these analyses do not provide evidence that loneliness‐related vmHRV reactivity patterns differed by sex; however, given the small male subsample, future studies with larger and more sex‐balanced samples are needed to further evaluate potential sex differences.

In the current study, mean levels of loneliness, perceived stress, and depressive symptoms were roughly comparable to those previously reported in other university student samples (e.g., Nadorff et al. 2011; Özdemir and Tuncay 2008; Roberti et al. 2006; Roddick and Chen 2021) and reflected moderate‐to‐high loneliness. This pattern is consistent with evidence that younger adults—particularly university students—tend to report elevated loneliness and psychological distress (Cacioppo and Patrick 2008; Ellard et al. 2023; Hawkley et al. 2022; Lee et al. 2021; Surkalim et al. 2022). At the same time, it is important to consider the context in which this study was conducted. Data collection occurred between January and April 2022, during a transitional phase of the COVID‐19 pandemic in which post‐secondary campuses in the region had reopened for in‐person research and instruction but continued to operate under hybrid learning and health‐safety measures. Although these conditions likely shaped individuals' social experiences, the direction and magnitude of their influence on loneliness were likely not uniform. Evidence from longitudinal and cross‐sectional studies indicates that university students' experiences of loneliness during this period were highly variable and potentially influenced by shifting public‐health restrictions, evolving learning environments, and access to in‐person and remote social opportunities (Amendola et al. 2024; Hu et al. 2024; Kambara et al. 2025; Towner et al. 2022; Truong et al. 2024). These transitional pandemic conditions may have contributed to variability in participants' social experiences and could help contextualize why structural social network indices, such as network size and diversity, were not associated with vmHRV reactivity in this sample. Future research will be needed to determine whether the findings observed in this study generalize to periods of more typical social engagement.

Although this study systematically examined vmHRV reactivity to cognitive demands—providing a necessary foundation in an area with limited prior research—it did not evaluate vmHRV recovery, another important component of physiological flexibility with implications for long‐term health (Laborde et al. 2018; Roddick et al. 2025). The tasks were intentionally designed as cognitively demanding but low‐threat challenges, rather than high‐arousal stressors, making post‐task recovery potentially less theoretically informative than in paradigms involving potent social or emotional demands. In addition, the study design included rest periods only after each full block of multiple tasks, rather than after each individual task, precluding a clear assessment of recovery dynamics. Moreover, during the rest period following the first block, participants would have anticipated a second round of cognitive challenge tasks, whereas the final rest period may have been influenced by fatigue or a desire to conclude the 2‐h session (Borchardt and Zoccola 2018; Roddick et al. 2025). Future studies would benefit from incorporating structured rest intervals after each task, particularly in the context of more socially salient or emotionally evocative stimuli, to enable a comprehensive evaluation of vmHRV recovery alongside reactivity.

A further methodological consideration is that the cognitive tasks were administered in a fixed order. Although the standardized sequence allowed for consistent comparisons across participants, it raises the possibility that order or fatigue effects could have influenced vmHRV responses. However, the lack of an association between chronic loneliness and vmHRV reactivity during the short‐term memory task—despite it appearing midway in the sequence—suggests that the primary findings are unlikely to have been driven by task order. Nonetheless, counterbalancing task presentation in future studies would provide a more rigorous test of potential sequence effects.

Another important direction for future research is to examine whether loneliness‐related blunting of vmHRV reactivity may reflect reduced effort mobilization or motivational disengagement. Motivational intensity theory proposes that cardiovascular responses during performance contexts vary as a function of effort mobilization. When task demands are perceived as manageable and success is valued, individuals should increase effort, indexed by heightened cardiovascular reactivity. In contrast, when tasks are perceived as too difficult or the required effort is not justified by the anticipated outcome, disengagement may occur, resulting in diminished cardiovascular reactivity (Brehm and Self 1989; Richter et al. 2016; Silvestrini 2017). In the present study, the cognitive tasks were intended as cognitively demanding but low‐threat challenges, and performance was generally high; however, effort and task motivation were not directly assessed. Future studies that directly assess both cardiovascular reactivity and motivational engagement would help clarify whether reduced effort mobilization or motivational disengagement contributes to loneliness‐related vmHRV blunting during cognitive demand.

Finally, although chronic loneliness was associated with blunted vmHRV responses to cognitive demand, loneliness did not reliably predict task performance. This dissociation raises the possibility that dysregulated vmHRV reactivity may emerge before measurable declines in cognitive functioning, potentially serving as an early physiological marker of loneliness‐related vulnerability. Given evidence that loneliness in older adults is linked to both cognitive and autonomic impairments (e.g., Guarnera et al. 2023; Hawkley and Cacioppo 2010; Quadt et al. 2020), future longitudinal studies could extend the present findings by examining whether blunted vmHRV reactivity to cognitive demands predicts subsequent cognitive decline. Such work would help clarify the role of altered neurovisceral regulation as a potential early indicator of loneliness‐related risk, and shed light on whether changes in cardiac vagal regulation precede, and perhaps contribute to, later cognitive impairments.

## 5 Conclusions

In summary, the present study offers evidence that chronic loneliness is linked to alterations in neurovisceral regulation, reflected in blunted vmHRV reactivity across diverse cognitive challenge tasks. Notably, these associations remained robust after accounting for a broad range of demographic, psychological, and health‐related factors, underscoring chronic loneliness as an independent correlate of reduced cardiac vagal flexibility under cognitive demand. An important strength of this study was the concurrent examination of chronic and situational loneliness, which enabled a clear comparison of their relative contributions to vmHRV reactivity. Although situational loneliness showed weaker and less consistent associations with vmHRV reactivity than chronic loneliness, exploratory analyses suggested that when elevated alongside chronic loneliness, it may further exacerbate physiological dysregulation. Together, these findings emphasize the value of distinguishing between temporal dimensions of loneliness and suggest that more persistent experiences of subjective social disconnection may become biologically embedded through disruptions in the coordination of cognitive and autonomic systems. This work highlights the importance of addressing loneliness—not only for psychological well‐being, but as a modifiable factor with implications for physiological systems that support adaptive cognitive function and long‐term health.

## Author Contributions

**Charlotte M. Roddick:** writing – original draft, project administration, investigation, formal analysis, software, validation, visualization, funding acquisition, conceptualization, data curation, methodology, resources, supervision, writing ‐ review and editing. **Yuxiao Wang:** formal analysis, data curation, investigation, software, validation, visualization, writing – review and editing. **Jiaying Zhao:** methodology, resources, supervision, writing – review and editing. **Lorne A. Whitehead:** data curation, funding acquisition, methodology, project administration, resources, writing – review and editing. **Jennifer A. Veitch:** methodology, writing – review and editing, resources. **Michael P. Royer:** funding acquisition, methodology, resources, writing – review and editing. **Dorukalp Durmus:** writing – review and editing, methodology. **Frances S. Chen:** conceptualization, data curation, methodology, resources, supervision, writing – review and editing.

## Funding

CMR was supported by a Canadian Social Sciences and Humanities Research Council Doctoral Award [6567] and a University of British Columbia Doctoral Fellowship [6456]. FSC was supported by a Michael Smith Health Research BC for Health Research Scholar Award [SCH‐2017‐2004]. The funders had no role in study design, data collection and analysis, decision to publish, or preparation of the manuscript.

## Conflicts of Interest

The authors declare no conflicts of interest.

## Supporting information


**Figure S1:** Overview of the study procedure. The figure depicts the approximately 30‐min adaptation period followed by one 45‐min block of rest periods and tasks. Each participant completed two of these 45‐min blocks, with a break between blocks. The 10‐min filler task was a neutrally valenced task in which participants rated their impressions of images of faces displaying neutral expressions. The 15‐min cognitive task battery included five tasks administered in a fixed order, assessing attention, perception, short‐term memory, working memory, and executive function. As part of a broader research project, each 45‐min block was administered under subtly different lighting conditions that were designed not to be visually apparent to participants, falling either outside the typical range of human visual detection or at its extreme edge. Lighting conditions were counterbalanced across participants. See the main text for details of the study procedure and the control of lighting condition and block order in analyses.
**Figure S2:** Predicted vagally mediated heart rate variability (vmHRV) reactivity to attention demands by levels of chronic loneliness, graphed separately for Block 1 and Block 2. VmHRV is quantified as the root mean square of successive differences (RMSSD), expressed in standardized units (*z*‐scores). Lines depict estimated vmHRV trajectories from linear mixed‐effects models at −1 SD, mean, and +1 SD of chronic loneliness (UCLA Loneliness Scale modified for a 5‐year timeframe; Russell 1996) to illustrate the significant association between higher chronic loneliness and blunted cardiac vagal withdrawal.
**Figure S3:** Predicted vagally mediated heart rate variability (vmHRV) reactivity to perception demands by levels of chronic loneliness, graphed separately for Block 1 and Block 2. VmHRV is quantified as the root mean square of successive differences (RMSSD), expressed in standardized units (*z*‐scores). Lines depict estimated vmHRV trajectories from linear mixed‐effects models at −1 SD, mean, and +1 SD of chronic loneliness (UCLA Loneliness Scale modified for a 5‐year timeframe; Russell 1996) to illustrate the significant association between higher chronic loneliness and blunted cardiac vagal withdrawal.
**Figure S4:** Predicted vagally mediated heart rate variability (vmHRV) reactivity to working memory demands by levels of chronic loneliness, graphed separately for Block 1 and Block 2. VmHRV is quantified as the root mean square of successive differences (RMSSD), expressed in standardized units (*z*‐scores). Lines depict estimated vmHRV trajectories from linear mixed‐effects models at −1 SD, mean, and +1 SD of chronic loneliness (UCLA Loneliness Scale modified for a 5‐year timeframe; Russell 1996) to illustrate the significant association between higher chronic loneliness and blunted cardiac vagal withdrawal.
**Figure S5:** Predicted vagally mediated heart rate variability (vmHRV) reactivity to executive function demands by levels of chronic loneliness, graphed separately for Block 1 and Block 2. VmHRV is quantified as the root mean square of successive differences (RMSSD), expressed in standardized units (*z*‐scores). Lines depict estimated vmHRV trajectories from linear mixed‐effects models at −1 SD, mean, and +1 SD of chronic loneliness (UCLA Loneliness Scale modified for a 5‐year timeframe; Russell 1996) to illustrate the significant association between higher chronic loneliness and blunted cardiac vagal withdrawal.
**Table S1:** Correlations between psychological and social network measures.
**Table S2:** Initial LMMs predicting vmHRV reactivity to cognitive tasks from situational loneliness.
**Table S3:** Covariate‐adjusted LMM predicting vmHRV reactivity to perception demands from situational loneliness.
**Table S4:** Covariate‐adjusted LMM predicting vmHRV reactivity to STM demands from situational loneliness.
**Table S5:** Covariate‐adjusted LMM predicting vmHRV reactivity to working memory demands from situational loneliness.
**Table S6:** Exploratory LMM predicting vmHRV reactivity to executive function demands from chronic and situational loneliness.
**Table S7:** Exploratory LMM predicting vmHRV reactivity to STM demands from chronic and situational loneliness.
**Table S8:** Exploratory LMM predicting vmHRV reactivity to attention demands from chronic and situational loneliness.
**Table S9:** Exploratory LMM predicting vmHRV reactivity to perception demands from chronic and situational loneliness.
**Table S10:** LMMs predicting vmHRV reactivity to cognitive tasks from chronic loneliness in Block 1.
**Table S11:** LMMs predicting vmHRV reactivity to cognitive tasks from chronic loneliness in Block 2.
**Table S12:** LMMs predicting vmHRV reactivity to cognitive tasks from situational loneliness in Block 1.
**Table S13:** LMMs predicting vmHRV reactivity to cognitive tasks from situational loneliness in Block 2.
**Table S14:** Linear mixed models predicting vmHRV reactivity to cognitive tasks from chronic loneliness (females only).
**Table S15:** Linear mixed models predicting vmHRV reactivity to cognitive tasks from chronic loneliness (males only).
**Table S16:** LMMs predicting vmHRV reactivity to cognitive tasks from situational loneliness (females only).
**Table S17:** LMMs predicting vmHRV reactivity to cognitive tasks from situational loneliness (males only).
**Table S18:** Linear mixed models predicting HF‐HRV reactivity to cognitive tasks from chronic loneliness.
**Table S19:** Linear mixed models predicting HF‐HRV reactivity to cognitive tasks from situational loneliness.
**Table S20:** Correlations between cognitive performance, vmHRV reactivity, and loneliness.

**Code S1:** R code used to conduct the main statistical analyses reported in the article.

**Dataset S1:** Dataset used to conduct the main statistical analyses reported in the article.

## Data Availability

The data and code for this manuscript are provided in the Supporting Information.

## Endnotes

^1^
Two participants were unable to complete the session due to late arrival or scheduling constraints.

^2^
In this task, participants were asked to rate their impressions of images of faces displaying neutral expressions.

^3^
Internal consistency metrics such as Cronbach's alpha are not applicable to the Social Network Index (Cohen et al. 1997) as the index captures the number and diversity of discrete social roles (e.g., friend, co‐worker, neighbor) rather than a unidimensional construct.

^4^
Data on gender were also collected; however, no participants reported a gender different from their assigned sex. Biological sex was used in analyses, as sex differences are known to influence vmHRV (Koenig and Thayer 2016; Quigley et al. 2024).

^5^
Further sensitivity analyses using high‐frequency heart rate variability (HF‐HRV, ln ms^2^) as an alternative metric of vmHRV reactivity revealed broadly similar directional patterns to those reported with RMSSD. However, these secondary analyses were statistically weaker, with significant associations observed primarily between chronic loneliness and HF‐HRV reactivity to the executive function task, and between situational loneliness and HF‐HRV reactivity to the short‐term memory task (see Tables S18 and S19 and summary of sensitivity analyses (HF‐HRV, ln ms^2^) in Supporting Information).

^6^
Neither chronic nor situational loneliness was significantly associated with cognitive task performance, with the exception that higher chronic loneliness was correlated with poorer performance on the perception task (*r* = −0.18, *p* = 0.031; see Table S20).
