# Supplementary material for: Loneliness as a Predictor of Blunted Heart Rate Variability Responses During Cognitive Demands
Source: Psychophysiology. 2026 Nov 13;63(11):e70432. doi: 10.1111/psyp.70432 (PMC13768148; doi:10.1111/psyp.70432)
Supplement: Supplementary file 1 [file PSYP-63-e70432-s001.docx]

**Supplemental Materials**

**Cognitive challenge tasks administered to participants (pp. 1–17)**

Task 1: Visual search

Task 2: Visual comparison

Task 3: Forward digit span

Task 4: Backward digit span

Task 5: Flanker

**Supplementary analyses (pp. 18–39)**

Tables S1 to S20

Summary of sensitivity analyses (HF-HRV, ln ms^2^)

Figures S1 to S5

**TASK 1: INSTRUCTIONS**

**1. Visual search task**

**Instructions:** On the following page, you will see a picture with many Ls and Ts in it. Your task is to **find and circle as many targets as you can** within the picture. The target is a **rotated T**, and there are a total of **50** targets. Once you find a target, you should circle it. You will have **90 seconds** to find and circle as many targets as possible. If you finish before the 90 seconds are up, please tell the experimenter that you have finished.

**PLEASE DO NOT TURN THE PAGE UNTIL YOU ARE INSTRUCTED TO DO SO**

**TASK 1**

Trial 1

Trial 2

**TASK 2: INSTRUCTIONS**

**2. Visual comparison task**

**Instructions:** This is an **example** of the second task. You will determine the differences between the two pictures below. Each time you find a difference, **circle the difference** **in the picture on the bottom**. Below is an example. You will have **2 minutes** to complete this task. If you finish before the 2 minutes are up, please tell the experimenter that you have finished.

**EXAMPLE:**

**PLEASE DO NOT TURN THE PAGE UNTIL YOU ARE INSTRUCTED TO DO SO**

**TASK 2**

Trial 1

Trial 2

**TASK 3**

**3. Short-term memory task**

**Instructions:** In this task, you will hear a series of digits. Please first try to memorize the digits, and then **recall the digits in the order in which they are heard**. For example, if you hear *3124*, please write down *3124*. For each trial, please hear the recording read out all the digits first, then recall the series by writing the digits down. Please recall the digits as accurately as you can. **The experimenter will only play each sequence once**. There are **10 trials** in total.

Trials (forward order)

1. ___	___	___

2. ___	___	___	___

3. ___	___	___	___	___

4. ___	___	___	___	___	___

5. ___	___	___	___	___	___	___

6. ___	___	___	___	___	___	___	___

7. ___	___	___	___	___	___	___	___	___

8. ___	___	___	___	___	___	___	___	___	___

9. ___	___	___	___	___	___	___	___	___	___	___

10. ___	___	___	___	___	___	___	___	___	___	___	___

**TASK 4**

**4. Working memory task**

**Instructions:** Like the previous task, you will hear a series of digits. Please first try to memorize the digits, and then **recall the digits in the reverse order**. For example, if you hear *3124*, please write down *4213*. For each trial, please listen to the recording read out all the digits first, then recall the sequence by writing the digits down. Please recall the digits as accurately as you can. **The experimenter will only play each sequence once**. There are **6 trials** in total.

Trials (reverse order)

1. ___	___	___

2. ___	___	___	___

3. ___	___	___	___	___

4. ___	___	___	___	___	___

5. ___	___	___	___	___	___	___

6. ___	___	___	___	___	___	___	___

**TASK 5 INSTRUCTIONS**

**5. Executive function task**

**Instructions:** This is the fourth task. You will see five arrows in a row, and your task is to **identify the direction of the central arrow** while ignoring the arrows on the left and right. In this example: → → ← → → the central arrow is pointing to the left, so you should circle L as your answer. In another example: → → → → → the central arrow is pointing to the right, so you should circle R as your answer. Please complete the following trials as quickly and accurately as you can. The experimenter will track how long it takes you to finish all trials. There are **100 trials** in total. When you have finished all 100 trials, inform the experimenter that you are done.

**EXAMPLE:**

→ → ← → →	L	R

**PLEASE DO NOT TURN THE PAGE UNTIL YOU ARE INSTRUCTED TO DO SO**

**TASK 5**

Trials

1. → → → → →	L	R

2. → → ← → →	L	R

3. ← ← → ← ←	L	R

4. ← ← ← ← ←	L	R

5. ← ← → ← ←	L	R

6. ← ← ← ← ←	L	R

7. → → → → →	L	R

8. → → ← → →	L	R

9. → → → → →	L	R

10. ← ← → ← ←	L	R

11. → → ← → →	L	R

12. ← ← ← ← ←	L	R

13. → → ← → →	L	R

14. ← ← → ← ←	L	R

15. → → → → →	L	R

16. ← ← ← ← ←	L	R

17. → → → → →	L	R

18. ← ← → ← ←	L	R

19. ← ← ← ← ←	L	R

20. → → ← → →	L	R

21. ← ← → ← ←	L	R

22. → → ← → →	L	R

23. ← ← ← ← ←	L	R

24. → → → → →	L	R

25. → → ← → →	L	R

26. ← ← ← ← ←	L	R

27. → → → → →	L	R

28. ← ← → ← ←	L	R

29. → → ← → →	L	R

30. ← ← → ← ←	L	R

31. → → → → →	L	R

32. ← ← ← ← ←	L	R

33. → → ← → →	L	R

34. → → → → →	L	R

35. ← ← → ← ←	L	R

36. ← ← ← ← ←	L	R

37. → → ← → →	L	R

38. ← ← → ← ←	L	R

39. → → → → →	L	R

40. ← ← ← ← ←	L	R

41. → → → → →	L	R

42. → → ← → →	L	R

43. ← ← ← ← ←	L	R

44. ← ← → ← ←	L	R

45. ← ← → ← ←	L	R

46. → → ← → →	L	R

47. → → → → →	L	R

48. ← ← ← ← ←	L	R

49. → → ← → →	L	R

50. → → → → →	L	R

51. ← ← → ← ←	L	R

52. ← ← ← ← ←	L	R

53. ← ← ← ← ←	L	R

54. ← ← → ← ←	L	R

55. → → ← → →	L	R

56. → → → → →	L	R

57. → → ← → →	L	R

58. ← ← ← ← ←	L	R

59. ← ← → ← ←	L	R

60. → → → → →	L	R

61. ← ← ← ← ←	L	R

62. → → → → →	L	R

63. ← ← → ← ←	L	R

64. → → ← → →	L	R

65. → → → → →	L	R

66. ← ← → ← ←	L	R

67. ← ← ← ← ←	L	R

68. → → ← → →	L	R

69. → → ← → →	L	R

70. ← ← → ← ←	L	R

71. ← ← ← ← ←	L	R

72. → → → → →	L	R

73. ← ← ← ← ←	L	R

74. → → ← → →	L	R

75. ← ← → ← ←	L	R

76. → → → → →	L	R

77. ← ← ← ← ←	L	R

78. → → → → →	L	R

79. → → ← → →	L	R

80. ← ← → ← ←	L	R

81. ← ← ← ← ←	L	R

82. → → ← → →	L	R

83. → → → → →	L	R

84. ← ← → ← ←	L	R

85. → → → → →	L	R

86. → → ← → →	L	R

87. ← ← ← ← ←	L	R

88. ← ← → ← ←	L	R

89. → → → → →	L	R

90. ← ← ← ← ←	L	R

91. → → ← → →	L	R

92. ← ← → ← ←	L	R

93. ← ← → ← ←	L	R

94. ← ← ← ← ←	L	R

95. → → ← → →	L	R

96. → → → → →	L	R

97. → → ← → →	L	R

98. → → → → →	L	R

99. ← ← ← ← ←	L	R

100. ← ← → ← ←	L	R

**Thank you. Please tell the experimenter that you have completed this task.**

**Supplementary analyses**

**Table S1.** *Correlations Between Psychological and Social Network Measures*

|  | chronic loneliness | situational loneliness | reappraisal | suppression | depression | anxiety | stress | network diversity | network size |
| --- | --- | --- | --- | --- | --- | --- | --- | --- | --- |
| chronic loneliness |  | 0.38^***^ | –0.14^†^ | 0.24^**^ | 0.43^***^ | 0.44^***^ | 0.39^***^ | –0.13 | –0.09 |
| situational loneliness |  |  | –0.15^†^ | 0.28^**^ | 0.58^***^ | 0.43^***^ | 0.56^***^ | –0.09 | –0.10 |
| reappraisal |  |  |  | –0.01 | –0.31^***^ | –0.24^**^ | –0.20^*^ | 0.07 | 0.02 |
| suppression |  |  |  |  | 0.29^***^ | 0.15^†^ | 0.34^***^ | –0.10 | –0.10 |
| depression |  |  |  |  |  | 0.77^***^ | 0.80^***^ | 0.01 | –0.03 |
| anxiety |  |  |  |  |  |  | 0.65^***^ | 0.04 | 0.04 |
| stress |  |  |  |  |  |  |  | –0.01 | –0.07 |
| network diversity |  |  |  |  |  |  |  |  | 0.26^**^ |
| network  size |  |  |  |  |  |  |  |  |  |

*Note*. ^†^ *p* < .10, ^*^ *p* < .05, ^**^ *p* < .01, ^***^ *p* < .001

**Table S2.** *Initial LMMs Predicting vmHRV Reactivity to Cognitive Tasks from Situational Loneliness*

|  |  | β | SE | *t* | *df* | 95% CI | *p* |
| --- | --- | --- | --- | --- | --- | --- | --- |
| **Attention** | |  |  |  |  |  |  |
|  | Period | 0.06 | 0.03 | 1.62 | 1650.05 | [–0.01, 0.12] | .106 |
|  | Block order | 0.40 | 0.03 | 13.82 | 1651.43 | [ 0.34, 0.46] | < .001^***^ |
|  | Situational loneliness | 0.01 | 0.07 | 0.18 | 153.26 | [–0.13, 0.15] | .858 |
|  | Block order × Period | –0.21 | 0.05 | –4.17 | 1650.13 | [–0.31, –0.11] | < .001^***^ |
|  | Situational loneliness × Period | 0.01 | 0.02 | 0.46 | 1650.22 | [–0.04, 0.06] | .646 |
| **Perception** | |  |  |  |  |  |  |
|  | Period | 0.25 | 0.03 | 8.59 | 1946.08 | [ 0.19, 0.31] | < .001^***^ |
|  | Block order | 0.40 | 0.03 | 14.54 | 1947.06 | [ 0.34, 0.45] | < .001^***^ |
|  | Situational loneliness | 0.01 | 0.07 | 0.17 | 153.70 | [–0.13, 0.15] | .865 |
|  | Block order × Period | –0.21 | 0.04 | –5.08 | 1946.12 | [–0.29, –0.13] | < .001^***^ |
|  | Situational loneliness × Period | 0.05 | 0.02 | 2.59 | 1946.21 | [ 0.01, 0.09] | .010^**^ |
| **Short-term memory** | |  |  |  |  |  |  |
|  | Period | 0.07 | 0.03 | 2.19 | 1788.61 | [0.01, 0.14] | .028^*^ |
|  | Block order | 0.42 | 0.03 | 13.91 | 1789.32 | [0.36, 0.47] | < .001^***^ |
|  | Situational loneliness | 0.01 | 0.07 | 0.19 | 154.14 | [–0.13, 0.15] | .851 |
|  | Block order × Period | –0.15 | 0.05 | –3.16 | 1788.36 | [–0.25, –0.06] | .002^**^ |
|  | Situational loneliness × Period | 0.05 | 0.02 | 2.18 | 1788.94 | [0.01, 0.10] | .029^*^ |
| **Working memory** | |  |  |  |  |  |  |
|  | Period | 0.02 | 0.04 | 0.42 | 1404.71 | [–0.06, 0.10] | .677 |
|  | Block order | 0.41 | 0.03 | 14.64 | 1405.51 | [0.36, 0.47] | < .001^***^ |
|  | Situational loneliness | 0.01 | 0.07 | 0.17 | 151.68 | [–0.13, 0.15] | .862 |
|  | Block order × Period | –0.16 | 0.06 | –2.71 | 1404.35 | [–0.27, –0.04] | .007^**^ |
|  | Situational loneliness × Period | 0.06 | 0.03 | 1.92 | 1404.92 | [–0.00, 0.12] | .055^†^ |
| **Executive function** | |  |  |  |  |  |  |
|  | Period | –0.08 | 0.04 | –2.22 | 1562.75 | [–0.15, –0.01] | .027^*^ |
|  | Block order | 0.41 | 0.03 | 14.61 | 1563.61 | [0.36, 0.47] | < .001^***^ |
|  | Situational loneliness | 0.01 | 0.07 | 0.18 | 152.74 | [–0.13, 0.15] | .857 |
|  | Block order × Period | –0.27 | 0.05 | –5.18 | 1562.47 | [–0.37, –0.17] | < .001^***^ |
|  | Situational loneliness × Period | 0.03 | 0.03 | 0.99 | 1563.47 | [–0.03, 0.08] | .322 |

*Note.* VmHRV is quantified using the root mean square of successive differences (RMSSD). vmHRV = vagally-mediated heart rate variability; LMM = linear mixed model; SE = standard error; CI = confidence interval. Period is coded 0 = baseline, 1 = task. Block order is coded 0 = first block, 1 = second block. ^*^ *p* < .05, ^**^ *p* < .01, ^***^ *p* < .001.

**Table S3.** *Covariate-Adjusted LMM Predicting vmHRV Reactivity to Perception Demands from Situational Loneliness*

|  | β | SE | *t* | *df* | 95% CI | *p* |
| --- | --- | --- | --- | --- | --- | --- |
| Period | 0.25 | 0.03 | 8.59 | 1944.07 | [0.19, 0.31] | < .001^***^ |
| Block order | 0.40 | 0.03 | 14.58 | 1945.11 | [0.34, 0.45] | < .001^***^ |
| Situational loneliness | 0.04 | 0.07 | 0.53 | 150.00 | [–0.10, 0.17] | .597 |
| Sex | 0.42 | 0.17 | 2.46 | 145.04 | [0.09, 0.74] | .015^*^ |
| Hours slept | –0.02 | 0.07 | –0.25 | 149.91 | [–0.15, 0.12] | .807 |
| Average caffeine consumption | 0.20 | 0.07 | 2.79 | 145.02 | [0.06, 0.34] | .006^**^ |
| Habitual cognitive reappraisal | 0.05 | 0.07 | 0.73 | 150.04 | [–0.08, 0.19] | .467 |
| Block order × Period | –0.21 | 0.04 | –5.03 | 1944.12 | [–0.29, –0.13] | < .001^***^ |
| Hours slept × Period | 0.05 | 0.02 | 2.35 | 1944.21 | [0.01, 0.09] | .019^*^ |
| Cognitive reappraisal × Period | –0.09 | 0.02 | –4.39 | 1944.34 | [–0.13, –0.05] | < .001^***^ |
| Situational loneliness × Period | 0.04 | 0.02 | 2.05 | 1944.28 | [0.00, 0.08] | .041^*^ |

*Note.* VmHRV is quantified using the root mean square of successive differences (RMSSD). vmHRV = vagally-mediated heart rate variability; LMM = linear mixed model; SE = standard error; CI = confidence interval. Period is coded 0 = baseline, 1 = task. Block order is coded 0 = first block, 1 = second block. Sex is coded 0 = male, 1 = female. ^*^ *p* < .05, ^**^ *p* < .01, ^***^ *p* < .001.

**Table S4.** *Covariate-Adjusted LMM Predicting vmHRV Reactivity to STM Demands from Situational Loneliness*

|  | β | SE | *t* | *df* | 95% CI | *p* |
| --- | --- | --- | --- | --- | --- | --- |
| Period | 0.07 | 0.03 | 2.18 | 1771.64 | [0.01, 0.14] | .029^*^ |
| Block order | 0.42 | 0.03 | 13.96 | 1772.36 | [0.36, 0.47] | < .001^***^ |
| Situational loneliness | 0.08 | 0.09 | 0.94 | 146.44 | [–0.08, 0.25] | .349 |
| Sex | 0.45 | 0.17 | 2.63 | 141.05 | [0.12, 0.78] | .010^**^ |
| Body mass index (BMI) | 0.00 | 0.07 | 0.04 | 145.88 | [–0.14, 0.14] | .968 |
| Hours slept | –0.03 | 0.07 | –0.38 | 146.09 | [–0.16, 0.11] | .705 |
| Average caffeine consumption | 0.21 | 0.08 | 2.80 | 145.74 | [0.07, 0.35] | .006^**^ |
| Perceived stress | –0.11 | 0.12 | –0.92 | 145.89 | [–0.33, 0.12] | .362 |
| Depression | 0.04 | 0.12 | 0.34 | 146.06 | [–0.19, 0.27] | .733 |
| Habitual cognitive reappraisal | 0.06 | 0.07 | 0.76 | 146.19 | [–0.08, 0.19] | .451 |
| Block order × Period | –0.15 | 0.05 | –3.17 | 1771.35 | [–0.25, –0.06] | .002^**^ |
| BMI × Period | –0.06 | 0.02 | –2.25 | 1771.69 | [–0.10, –0.01] | .025^*^ |
| Hours slept × Period | 0.07 | 0.02 | 2.91 | 1771.88 | [0.02, 0.12] | .004^**^ |
| Average caffeine × Period | –0.08 | 0.03 | –3.07 | 1772.00 | [–0.13, –0.03] | .002^**^ |
| Perceived stress × Period | 0.08 | 0.04 | 2.03 | 1771.66 | [0.00, 0.16] | .042^*^ |
| Depression × Period | 0.00 | 0.04 | 0.02 | 1771.85 | [–0.08, 0.08] | .988 |
| Cognitive reappraisal × Period | –0.05 | 0.03 | –1.80 | 1772.40 | [–0.10, 0.00] | .072^†^ |
| Situational loneliness × Period | 0.00 | 0.03 | 0.04 | 1772.36 | [–0.06, 0.06] | .965 |

*Note.* VmHRV is quantified using the root mean square of successive differences (RMSSD). vmHRV = vagally-mediated heart rate variability; STM = short-term memory; LMM = linear mixed model; SE = standard error; CI = confidence interval. Period is coded 0 = baseline, 1 = task. Block order is coded 0 = first block, 1 = second block. Sex is coded 0 = male, 1 = female. ^†^ *p* < .10, ^*^ *p* < .05, ^**^ *p* < .01, ^***^ *p* < .001.

**Table S5.** *Covariate-Adjusted LMM Predicting vmHRV Reactivity to Working Memory Demands from Situational Loneliness*

|  | β | SE | *t* | *df* | 95% CI | *p* |
| --- | --- | --- | --- | --- | --- | --- |
| Period | 0.02 | 0.04 | 0.44 | 1401.72 | [–0.06, 0.10] | .661 |
| Block order | 0.41 | 0.03 | 14.68 | 1402.58 | [0.36, 0.47] | < .001^***^ |
| Situational loneliness | 0.07 | 0.09 | 0.77 | 147.09 | [–0.10, 0.23] | .443 |
| Sex | 0.45 | 0.17 | 2.64 | 144.18 | [0.12, 0.78] | .009^**^ |
| Hours slept | –0.02 | 0.07 | –0.34 | 146.72 | [–0.16, 0.11] | .732 |
| Average caffeine consumption | 0.18 | 0.07 | 2.56 | 144.00 | [0.04, 0.32] | .012^*^ |
| Perceived stress | –0.10 | 0.12 | –0.90 | 146.59 | [–0.33, 0.12] | .371 |
| Depression | 0.04 | 0.12 | 0.32 | 146.77 | [–0.19, 0.26] | .753 |
| Block order × Period | –0.16 | 0.06 | –2.75 | 1401.33 | [–0.27, –0.05] | .006^**^ |
| Hours slept × Period | 0.08 | 0.03 | 2.87 | 1402.17 | [0.03, 0.14] | .004^**^ |
| Perceived stress × Period | 0.07 | 0.05 | 1.44 | 1401.72 | [–0.03, 0.17] | .151 |
| Depression × Period | 0.00 | 0.05 | 0.09 | 1401.94 | [–0.09, 0.10] | .927 |
| Situational loneliness × Period | 0.02 | 0.04 | 0.56 | 1402.31 | [–0.05, 0.09] | .573 |

*Note.* VmHRV is quantified using the root mean square of successive differences (RMSSD). vmHRV = vagally-mediated heart rate variability; LMM = linear mixed model; SE = standard error; CI = confidence interval. Period is coded 0 = baseline, 1 = task. Block order is coded 0 = first block, 1 = second block. Sex is coded 0 = male, 1 = female. ^*^ *p* < .05, ^**^ *p* < .01, ^***^ *p* < .001.

| **Table S6**. *Exploratory LMM Predicting vmHRV Reactivity to Executive Function Demands from Chronic and Situational Loneliness* | | | | | | |
| --- | --- | --- | --- | --- | --- | --- |
|  | β | SE | *t* | *df* | 95% CI | *p* |
| Period | –0.10 | 0.04 | –2.74 | 1560.78 | [–0.17, –0.03] | .006^**^ |
| Block order | 0.41 | 0.03 | 14.62 | 1561.62 | [0.36, 0.47] | < .001^***^ |
| Chronic loneliness | 0.11 | 0.08 | 1.38 | 150.66 | [–0.04, 0.25] | .170 |
| Situational loneliness | –0.03 | 0.08 | –0.41 | 150.80 | [–0.18, 0.12] | .684 |
| Block order × Period | –0.27 | 0.05 | –5.16 | 1560.49 | [–0.37, –0.17] | < .001^***^ |
| Chronic loneliness × Period | 0.07 | 0.03 | 2.63 | 1561.08 | [0.02, 0.13] | .009^**^ |
| Situational loneliness × Period | 0.00 | 0.03 | –0.01 | 1561.47 | [–0.06, 0.05] | .993 |
| Chronic loneliness × Situational loneliness | –0.07 | 0.07 | –0.93 | 150.67 | [–0.21, 0.08] | .355 |
| Chronic × Situational × Period | 0.05 | 0.03 | 1.96 | 1561.04 | [–0.00, 0.10] | .051^†^ |

*Note.* VmHRV is quantified using the root mean square of successive differences (RMSSD). vmHRV = vagally-mediated heart rate variability; LMM = linear mixed model; SE = standard error; CI = confidence interval. Period is coded 0 = baseline, 1 = task. Block order is coded 0 = first block, 1 = second block. ^†^ *p* < .10, ^*^ *p* < .05, ^**^ *p* < .01, ^***^ *p* < .001.

**Table S7.** *Exploratory LMM Predicting vmHRV Reactivity to STM Demands from Chronic and Situational Loneliness*

|  | β | SE | *t* | *df* | 95% CI | *p* |
| --- | --- | --- | --- | --- | --- | --- |
| Period | 0.06 | 0.04 | 1.59 | 1786.66 | [–0.01, 0.13] | .113 |
| Block order | 0.42 | 0.03 | 13.90 | 1787.31 | [0.36, 0.47] | < .001^***^ |
| Chronic loneliness | 0.11 | 0.08 | 1.39 | 152.03 | [–0.04, 0.25] | .168 |
| Situational loneliness | –0.03 | 0.08 | –0.41 | 152.17 | [–0.18, 0.12] | .686 |
| Block order × Period | –0.15 | 0.05 | –3.14 | 1786.35 | [–0.25, –0.06] | .002^**^ |
| Chronic loneliness × Period | 0.02 | 0.03 | 0.64 | 1786.86 | [–0.03, 0.07] | .524 |
| Situational loneliness × Period | 0.05 | 0.03 | 1.83 | 1787.12 | [–0.00, 0.10] | .068^†^ |
| Chronic loneliness × Situational loneliness | –0.07 | 0.07 | –0.93 | 152.04 | [–0.21, 0.08] | .353 |
| Chronic × Situational × Period | 0.04 | 0.03 | 1.67 | 1786.79 | [–0.01, 0.09] | .095^†^ |

*Note.* VmHRV is quantified using the root mean square of successive differences (RMSSD). vmHRV = vagally-mediated heart rate variability; STM = short-term memory; LMM = linear mixed model; SE = standard error; CI = confidence interval.

Period is coded 0 = baseline, 1 = task. Block order is coded 0 = first block, 1 = second block. ^†^ *p* < .10, ^*^ *p* < .05, ^**^ *p* < .01, ^***^ *p* < .001.

**Table S8.** *Exploratory LMM Predicting vmHRV Reactivity to Attention Demands from Chronic and Situational Loneliness*

|  | β | SE | *t* | *df* | 95% CI | *p* |
| --- | --- | --- | --- | --- | --- | --- |
| Period | 0.05 | 0.04 | 1.33 | 1648.07 | [–0.02, 0.12] | .184 |
| Block order | 0.40 | 0.03 | 13.82 | 1649.43 | [0.34, 0.46] | < .001^***^ |
| Chronic loneliness | 0.10 | 0.08 | 1.34 | 151.17 | [–0.05, 0.25] | .183 |
| Situational loneliness | –0.03 | 0.08 | –0.39 | 151.31 | [–0.18, 0.12] | .697 |
| Block order × Period | –0.21 | 0.05 | –4.13 | 1648.14 | [–0.30, –0.11] | < .001^***^ |
| Chronic loneliness × Period | 0.07 | 0.03 | 2.66 | 1648.13 | [0.02, 0.12] | .008^**^ |
| Situational loneliness × Period | –0.02 | 0.03 | –0.57 | 1648.30 | [–0.07, 0.04] | .571 |
| Chronic loneliness × Situational loneliness | –0.07 | 0.07 | –0.90 | 151.18 | [–0.21, 0.08] | .370 |
| Chronic × Situational × Period | 0.02 | 0.03 | 0.72 | 1648.14 | [–0.03, 0.07] | .473 |

*Note.* VmHRV is quantified using the root mean square of successive differences (RMSSD). vmHRV = vagally-mediated heart rate variability; LMM = linear mixed model; SE = standard error; CI = confidence interval. Period is coded 0 = baseline, 1 = task. Block order is coded 0 = first block, 1 = second block. ^*^ *p* < .05, ^**^ *p* < .01, ^***^ *p* < .001.

**Table S9.** *Exploratory LMM Predicting vmHRV Reactivity to Perception Demands from Chronic and Situational Loneliness*

|  | β | SE | *t* | *df* | 95% CI | *p* |
| --- | --- | --- | --- | --- | --- | --- |
| Period | 0.25 | 0.03 | 8.39 | 1944.10 | [0.20, 0.31] | < .001^***^ |
| Block order | 0.40 | 0.03 | 14.54 | 1945.06 | [0.34, 0.45] | < .001^***^ |
| Chronic loneliness | 0.10 | 0.08 | 1.29 | 151.62 | [–0.05, 0.25] | .198 |
| Situational loneliness | –0.03 | 0.08 | –0.38 | 151.74 | [–0.18, 0.12] | .705 |
| Block order × Period | –0.21 | 0.04 | –5.07 | 1944.12 | [–0.29, –0.13] | < .001^***^ |
| Chronic loneliness × Period | 0.04 | 0.02 | 1.78 | 1944.11 | [–0.00, 0.08] | .076^†^ |
| Situational loneliness × Period | 0.04 | 0.02 | 1.67 | 1944.29 | [–0.01, 0.08] | .095^†^ |
| Chronic loneliness × Situational loneliness | –0.07 | 0.08 | –0.87 | 151.63 | [–0.21, 0.08] | .386 |
| Chronic × Situational × Period | –0.01 | 0.02 | –0.58 | 1944.13 | [–0.06, 0.03] | .564 |

*Note.* VmHRV is quantified using the root mean square of successive differences (RMSSD). vmHRV = vagally-mediated heart rate variability; LMM = linear mixed model; SE = standard error; CI = confidence interval. Period is coded 0 = baseline, 1 = task. Block order is coded 0 = first block, 1 = second block. ^†^ *p* < .10, ^*^ *p* < .05, ^**^ *p* < .01, ^***^ *p* < .001.

**Table S10**. *LMMs Predicting vmHRV Reactivity to Cognitive Tasks from Chronic Loneliness in Block 1*

|  |  | β | SE | *t* | *df* | 95% CI | *p* |
| --- | --- | --- | --- | --- | --- | --- | --- |
| **Attention** | |  |  |  |  |  |  |
|  | Period | 0.06 | 0.03 | 1.93 | 770.26 | [–0.00, 0.11] | .054^†^ |
|  | Chronic loneliness | 0.08 | 0.06 | 1.23 | 156.59 | [–0.05, 0.20] | .220 |
|  | Chronic loneliness × Period | 0.07 | 0.03 | 2.38 | 770.25 | [0.01, 0.13] | .018^*^ |
| **Perception** | |  |  |  |  |  |  |
|  | Period | 0.25 | 0.02 | 10.04 | 918.32 | [0.20, 0.30] | < .001^***^ |
|  | Chronic loneliness | 0.07 | 0.06 | 1.13 | 157.65 | [–0.05, 0.20] | .261 |
|  | Chronic loneliness × Period | 0.10 | 0.02 | 3.85 | 918.26 | [0.05, 0.14] | < .001^***^ |
| **Short-term memory** | |  |  |  |  |  |  |
|  | Period | 0.08 | 0.03 | 2.81 | 841.62 | [0.02, 0.14] | .005^**^ |
|  | Chronic loneliness | 0.08 | 0.06 | 1.26 | 158.99 | [–0.04, 0.20] | .208 |
|  | Chronic loneliness × Period | 0.02 | 0.03 | 0.61 | 841.35 | [–0.04, 0.07] | .544 |
| **Working memory** | |  |  |  |  |  |  |
|  | Period | 0.01 | 0.03 | 0.31 | 650.42 | [–0.05, 0.07] | .756 |
|  | Chronic loneliness | 0.07 | 0.06 | 1.21 | 153.84 | [–0.05, 0.20] | .228 |
|  | Chronic loneliness × Period | 0.08 | 0.03 | 2.51 | 650.25 | [0.02, 0.15] | .012^*^ |
| **Executive function** | |  |  |  |  |  |  |
|  | Period | –0.07 | 0.03 | –2.56 | 740.19 | [–0.13, –0.02] | .011^*^ |
|  | Chronic loneliness | 0.08 | 0.06 | 1.21 | 155.34 | [–0.05, 0.20] | .227 |
|  | Chronic loneliness × Period | 0.05 | 0.03 | 1.91 | 739.99 | [–0.00, 0.11] | .056^†^ |

*Note.* VmHRV is quantified using the root mean square of successive differences (RMSSD). vmHRV = vagally-mediated heart rate variability; LMM = linear mixed model; SE = standard error; CI = confidence interval. Period is coded 0 = baseline, 1 = task.

^†^ *p* < .10, ^*^ *p* < .05, ^**^ *p* < .01, ^***^ *p* < .001.

**Table S11.** *LMMs Predicting vmHRV Reactivity to Cognitive Tasks from Chronic Loneliness in Block 2*

|  |  | β | SE | *t* | *df* | 95% CI | *p* |
| --- | --- | --- | --- | --- | --- | --- | --- |
| **Attention** | |  |  |  |  |  |  |
|  | Period | –0.15 | 0.04 | –4.25 | 732.91 | [–0.22, –0.08] | < .001^***^ |
|  | Chronic loneliness | 0.11 | 0.08 | 1.26 | 151.92 | [–0.06, 0.27] | .209 |
|  | Chronic loneliness × Period | 0.06 | 0.04 | 1.70 | 732.32 | [–0.01, 0.13] | .089^†^ |
| **Perception** | |  |  |  |  |  |  |
|  | Period | 0.04 | 0.03 | 1.38 | 880.57 | [–0.02, 0.10] | .167 |
|  | Chronic loneliness | 0.10 | 0.08 | 1.25 | 152.94 | [–0.06, 0.27] | .213 |
|  | Chronic loneliness × Period | 0.01 | 0.03 | 0.49 | 880.20 | [–0.04, 0.07] | .627 |
| **Short-term memory** | |  |  |  |  |  |  |
|  | Period | –0.07 | 0.03 | –2.16 | 801.23 | [–0.14, –0.01] | .031^*^ |
|  | Chronic loneliness | 0.11 | 0.08 | 1.28 | 152.86 | [–0.06, 0.27] | .202 |
|  | Chronic loneliness × Period | 0.05 | 0.03 | 1.56 | 800.68 | [–0.01, 0.12] | .119 |
| **Working memory** | |  |  |  |  |  |  |
|  | Period | –0.14 | 0.04 | –3.46 | 608.62 | [–0.22, –0.06] | < .001^***^ |
|  | Chronic loneliness | 0.11 | 0.09 | 1.26 | 149.18 | [–0.06, 0.27] | .210 |
|  | Chronic loneliness × Period | 0.12 | 0.04 | 3.04 | 608.00 | [0.04, 0.20] | .002^**^ |
| **Executive function** | |  |  |  |  |  |  |
|  | Period | –0.36 | 0.04 | –9.54 | 677.43 | [–0.44, –0.29] | < .001^***^ |
|  | Chronic loneliness | 0.11 | 0.08 | 1.31 | 151.34 | [–0.05, 0.27] | .192 |
|  | Chronic loneliness × Period | 0.09 | 0.04 | 2.30 | 676.71 | [0.01, 0.16] | .022^*^ |

*Note.* VmHRV is quantified using the root mean square of successive differences (RMSSD). vmHRV = vagally-mediated heart rate variability; LMM = linear mixed model; SE = standard error; CI = confidence interval. Period is coded 0 = baseline, 1 = task.

^†^ *p* < .10, ^*^ *p* < .05, ^**^ *p* < .01, ^***^ *p* < .001.

**Table S12.**

*LMMs Predicting vmHRV Reactivity to Cognitive Tasks from Situational Loneliness in Block 1*

|  |  | β | SE | *t* | *df* | 95% CI | *p* |
| --- | --- | --- | --- | --- | --- | --- | --- |
| **Attention** | |  |  |  |  |  |  |
|  | Period | 0.06 | 0.03 | 1.96 | 770.29 | [–0.00, 0.11] | .051^†^ |
|  | Situational loneliness | 0.02 | 0.06 | 0.30 | 156.11 | [–0.10, 0.14] | .765 |
|  | Situational loneliness × Period | 0.02 | 0.03 | 0.66 | 770.26 | [–0.04, 0.07] | .511 |
| **Perception** | |  |  |  |  |  |  |
|  | Period | 0.25 | 0.03 | 9.99 | 918.34 | [0.20, 0.30] | < .001^***^ |
|  | Situational loneliness | 0.02 | 0.06 | 0.26 | 157.19 | [–0.11, 0.14] | .794 |
|  | Situational loneliness × Period | 0.05 | 0.02 | 2.00 | 918.38 | [0.00, 0.10] | .046^*^ |
| **Short-term memory** | |  |  |  |  |  |  |
|  | Period | 0.08 | 0.03 | 2.80 | 841.62 | [ 0.02, 0.14] | .005^**^ |
|  | Situational loneliness | 0.02 | 0.06 | 0.30 | 158.35 | [–0.10, 0.14] | .765 |
|  | Situational loneliness × Period | 0.02 | 0.03 | 0.76 | 841.21 | [–0.03, 0.08] | .445 |
| **Working memory** | |  |  |  |  |  |  |
|  | Period | 0.01 | 0.03 | 0.33 | 650.38 | [–0.05, 0.08] | .738 |
|  | Situational loneliness | 0.02 | 0.06 | 0.27 | 153.44 | [–0.10, 0.14] | .784 |
|  | Situational loneliness × Period | 0.04 | 0.03 | 1.29 | 650.17 | [–0.02, 0.11] | .197 |
| **Executive function** | |  |  |  |  |  |  |
|  | Period | –0.07 | 0.03 | –2.51 | 740.19 | [–0.13, –0.02] | .013^*^ |
|  | Situational loneliness | 0.02 | 0.06 | 0.28 | 154.94 | [–0.11, 0.14] | .782 |
|  | Situational loneliness × Period | 0.02 | 0.03 | 0.86 | 740.27 | [–0.03, 0.08] | .390 |

*Note.* VmHRV is quantified using the root mean square of successive differences (RMSSD). vmHRV = vagally-mediated heart rate variability; LMM = linear mixed model; SE = standard error; CI = confidence interval. Period is coded 0 = baseline, 1 = task.

^†^ *p* < .10, ^*^ *p* < .05, ^**^ *p* < .01, ^***^ *p* < .001.

**Table S13.**

*LMMs Predicting vmHRV Reactivity to Cognitive Tasks from Situational Loneliness in Block 2*

|  |  | β | SE | *t* | *df* | 95% CI | *p* |
| --- | --- | --- | --- | --- | --- | --- | --- |
| **Attention** | |  |  |  |  |  |  |
|  | Period | –0.15 | 0.04 | –4.28 | 732.84 | [–0.22, –0.08] | < .001^***^ |
|  | Situational loneliness | 0.00 | 0.09 | 0.04 | 152.47 | [–0.16, 0.17] | .970 |
|  | Situational loneliness × Period | –0.00 | 0.04 | –0.00 | 733.73 | [–0.07, 0.07] | .997 |
| **Perception** | |  |  |  |  |  |  |
|  | Period | 0.04 | 0.03 | 1.41 | 880.53 | [–0.02, 0.10] | .159 |
|  | Situational loneliness | 0.00 | 0.08 | 0.03 | 153.30 | [–0.16, 0.17] | .978 |
|  | Situational loneliness × Period | 0.06 | 0.03 | 1.85 | 881.10 | [–0.00, 0.11] | .065^†^ |
| **Short-term memory** | |  |  |  |  |  |  |
|  | Period | –0.07 | 0.03 | –2.08 | 801.13 | [–0.14, –0.00] | .038^*^ |
|  | Situational loneliness | 0.00 | 0.09 | 0.04 | 153.26 | [–0.17, 0.17] | .971 |
|  | Situational loneliness × Period | 0.09 | 0.03 | 2.71 | 801.89 | [ 0.03, 0.16] | .007^**^ |
| **Working memory** | |  |  |  |  |  |  |
|  | Period | –0.14 | 0.04 | –3.41 | 608.67 | [–0.22, –0.06] | < .001^***^ |
|  | Situational loneliness | 0.00 | 0.09 | 0.01 | 149.88 | [–0.17, 0.17] | .988 |
|  | Situational loneliness × Period | 0.08 | 0.04 | 1.83 | 609.69 | [–0.01, 0.16] | .068^†^ |
| **Executive function** | |  |  |  |  |  |  |
|  | Period | –0.36 | 0.04 | –9.45 | 677.46 | [–0.44, –0.29] | < .001^***^ |
|  | Situational loneliness | 0.00 | 0.08 | 0.04 | 152.12 | [–0.16, 0.17] | .968 |
|  | Situational loneliness × Period | 0.02 | 0.04 | 0.44 | 678.39 | [–0.06, 0.09] | .658 |

*Note.* VmHRV is quantified using the root mean square of successive differences (RMSSD). vmHRV = vagally-mediated heart rate variability; LMM = linear mixed model; SE = standard error; CI = confidence interval. Period is coded 0 = baseline, 1 = task.

^†^ *p* < .10, ^*^ *p* < .05, ^**^ *p* < .01, ^***^ *p* < .001.

**Table S14.**

*Linear Mixed Models Predicting vmHRV Reactivity to Cognitive Tasks from Chronic Loneliness (Females Only)*

|  |  | β | SE | *t* | *df* | 95% CI | *p* |
| --- | --- | --- | --- | --- | --- | --- | --- |
| **Attention** | |  |  |  |  |  |  |
|  | Period | 0.04 | 0.04 | 1.12 | 1282.02 | [–0.03, 0.12] | .264 |
|  | Chronic loneliness | 0.47 | 0.03 | 14.33 | 1283.18 | [0.41, 0.53] | < .001^***^ |
|  | Block order | 0.15 | 0.08 | 1.88 | 118.16 | [–0.01, 0.31] | .063 |
|  | Block order × Period | –0.26 | 0.06 | –4.67 | 1282.01 | [–0.37, –0.15] | < .001^***^ |
|  | Chronic loneliness × Period | 0.08 | 0.03 | 2.78 | 1282.01 | [0.02, 0.13] | .006^**^ |
| **Perception** | |  |  |  |  |  |  |
|  | Period | 0.26 | 0.03 | 8.43 | 1509.07 | [0.22, 0.35] | < .001^***^ |
|  | Chronic loneliness | 0.47 | 0.03 | 14.64 | 1509.94 | [0.40, 0.53] | < .001^***^ |
|  | Block order | 0.15 | 0.08 | 1.80 | 118.70 | [–0.01, 0.31] | .074 |
|  | Block order × Period | –0.30 | 0.05 | –6.15 | 1509.04 | [–0.39, –0.20] | < .001^***^ |
|  | Chronic loneliness × Period | 0.05 | 0.02 | 2.03 | 1509.03 | [0.00, 0.10] | .043^*^ |
| **Short-term memory** | |  |  |  |  |  |  |
|  | Period | 0.08 | 0.04 | 2.13 | 1390.49 | [0.01, 0.16] | .034^*^ |
|  | Chronic loneliness | 0.49 | 0.04 | 13.97 | 1391.24 | [0.42, 0.56] | < .001^***^ |
|  | Block order | 0.16 | 0.08 | 1.95 | 119.14 | [–0.00, 0.32] | .053 |
|  | Block order × Period | –0.23 | 0.06 | –3.98 | 1390.30 | [–0.34, –0.11] | < .001^***^ |
|  | Chronic loneliness × Period | 0.03 | 0.03 | 0.91 | 1390.55 | [–0.03, 0.08] | .363 |
| **Working memory** | |  |  |  |  |  |  |
|  | Period | 0.03 | 0.05 | 0.54 | 1093.63 | [–0.07, 0.12] | .591 |
|  | Chronic loneliness | 0.48 | 0.03 | 14.79 | 1094.28 | [0.42, 0.55] | < .001^***^ |
|  | Block order | 0.15 | 0.08 | 1.90 | 117.12 | [–0.01, 0.31] | .060 |
|  | Block order × Period | –0.23 | 0.07 | –3.26 | 1093.29 | [–0.36, –0.10] | < .001^***^ |
|  | Chronic loneliness × Period | 0.11 | 0.03 | 3.26 | 1093.78 | [0.04, 0.18] | .001^**^ |
| **Executive function** | |  |  |  |  |  |  |
|  | Period | –0.08 | 0.04 | –2.01 | 1227.54 | [–0.16, –0.00] | .044^*^ |
|  | Chronic loneliness | 0.49 | 0.03 | 14.78 | 1228.44 | [0.42, 0.55] | < .001^***^ |
|  | Block order | 0.16 | 0.08 | 1.93 | 118.06 | [–0.00, 0.32] | .056 |
|  | Block order × Period | –0.34 | 0.06 | –5.72 | 1227.41 | [–0.46, –0.22] | < .001^***^ |
|  | Chronic loneliness × Period | 0.07 | 0.03 | 2.38 | 1227.69 | [0.01, 0.13] | .018^**^ |

*Note.* *n* = 117 females. VmHRV is quantified using the root mean square of successive differences (RMSSD). vmHRV = vagally-mediated heart rate variability; SE = standard error; CI = confidence interval. Period is coded 0 = baseline, 1 = task. Block order is coded 0 = first block, 1 = second block. ^*^ *p* < .05, ^**^ *p* < .01, ^***^ *p* < .001.

**Table S15.**

*Linear Mixed Models Predicting vmHRV Reactivity to Cognitive Tasks from Chronic Loneliness*

*(Males Only)*

|  |  | β | SE | *t* | *df* | 95% CI | *p* |
| --- | --- | --- | --- | --- | --- | --- | --- |
| **Attention** | |  |  |  |  |  |  |
|  | Period | 0.09 | 0.07 | 1.30 | 364.03 | [–0.05, 0.23] | .195 |
|  | Chronic loneliness | 0.16 | 0.06 | 2.68 | 364.35 | [0.04, 0.27] | .008^**^ |
|  | Block order | –0.10 | 0.12 | –0.79 | 33.31 | [–0.32, 0.14] | .436 |
|  | Block order × Period | –0.001 | 0.10 | –0.02 | 364.17 | [–0.20, 0.20] | .985 |
|  | Chronic loneliness × Period | 0.02 | 0.05 | 0.42 | 364.06 | [–0.08, 0.12] | .674 |
| **Perception** | |  |  |  |  |  |  |
|  | Period | 0.13 | 0.05 | 2.37 | 433.01 | [0.02, 0.23] | .018^*^ |
|  | Chronic loneliness | 0.16 | 0.05 | 3.13 | 33.27 | [0.06, 0.26] | .002^**^ |
|  | Block order | –0.10 | 0.11 | –0.79 | 433.19 | [–0.32, 0.13] | .437 |
|  | Block order × Period | 0.09 | 0.08 | 1.16 | 433.10 | [–0.06, 0.24] | .245 |
|  | Chronic loneliness × Period | 0.07 | 0.04 | 2.01 | 433.03 | [0.00, 0.15] | .045^*^ |
| **Short-term memory** | |  |  |  |  |  |  |
|  | Period | 0.05 | 0.06 | 0.75 | 394.16 | [–0.07, 0.17] | .453 |
|  | Chronic loneliness | 0.16 | 0.05 | 3.04 | 394.21 | [0.06, 0.27] | .003^**^ |
|  | Block order | –0.10 | 0.11 | –0.83 | 33.26 | [–0.32, 0.13] | .411 |
|  | Block order × Period | 0.09 | 0.09 | 0.99 | 394.12 | [–0.08, 0.25] | .325 |
|  | Chronic loneliness × Period | 0.05 | 0.04 | 1.17 | 394.21 | [–0.03, 0.13] | .243 |
| **Working memory** | |  |  |  |  |  |  |
|  | Period | –0.01 | 0.08 | –0.18 | 307.12 | [–0.16, 0.13] | .856 |
|  | Chronic loneliness | 0.16 | 0.05 | 3.21 | 307.33 | [0.06, 0.26] | .001^**^ |
|  | Block order | –0.09 | 0.12 | –1.80 | 32.59 | [–0.32, 0.13] | .428 |
|  | Block order × Period | 0.08 | 0.11 | 0.71 | 307.08 | [–0.13, 0.29] | .481 |
|  | Chronic loneliness × Period | 0.08 | 0.05 | 1.43 | 307.21 | [–0.03, 0.18] | .154 |
| **Executive function** | |  |  |  |  |  |  |
|  | Period | –0.06 | 0.07 | –0.91 | 331.14 | [–0.20, 0.07] | .366 |
|  | Chronic loneliness | 0.16 | 0.05 | 3.17 | 331.19 | [0.06, 0.26] | .002^**^ |
|  | Block order | –0.09 | 0.12 | –0.82 | 32.68 | [–0.32, 0.13] | .420 |
|  | Block order × Period | –0.02 | 0.10 | –0.20 | 330.99 | [–0.21, 0.17] | .843 |
|  | Chronic loneliness × Period | 0.06 | 0.05 | 1.30 | 331.31 | [–0.03, 0.15] | .195 |

*Note.* *n* = 34 males. VmHRV is quantified using the root mean square of successive differences (RMSSD). vmHRV = vagally-mediated heart rate variability; SE = standard error; CI = confidence interval. Period is coded 0 = baseline, 1 = task. Block order is coded 0 = first block, 1 = second block. ^*^ *p* < .05, ^**^ *p* < .01, ^***^ *p* < .001.

**Table S16.**

*LMMs Predicting vmHRV Reactivity to Cognitive Tasks from Situational Loneliness (Females Only)*

|  |  | β | SE | *t* | *df* | 95% CI | *p* |
| --- | --- | --- | --- | --- | --- | --- | --- |
| **Attention** | |  |  |  |  |  |  |
|  | Period | 0.05 | 0.04 | 1.18 | 1282.02 | [–0.03, 0.12] | .240 |
|  | Situational loneliness | 0.47 | 0.03 | 14.31 | 1283.12 | [0.41, 0.54] | < .001^***^ |
|  | Block order | 0.07 | 0.08 | 0.86 | 118.08 | [–0.09, 0.23] | .390 |
|  | Block order × Period | –0.27 | 0.06 | –4.72 | 1282.01 | [–0.38, –0.16] | < .001^***^ |
|  | Situational loneliness × Period | 0.00 | 0.03 | 0.08 | 1282.01 | [–0.05, 0.06] | .935 |
| **Perception** | |  |  |  |  |  |  |
|  | Period | 0.29 | 0.03 | 8.46 | 1509.06 | [ 0.22, 0.35] | < .001^***^ |
|  | Situational loneliness | 0.47 | 0.03 | 14.62 | 1509.89 | [0.40, 0.53] | < .001^***^ |
|  | Block order | 0.07 | 0.08 | 0.83 | 118.60 | [–0.09, 0.23] | .406 |
|  | Block order × Period | –0.30 | 0.05 | –6.11 | 1509.04 | [–0.39, –0.20] | < .001^***^ |
|  | Situational loneliness × Period | 0.05 | 0.02 | 2.19 | 1509.05 | [ 0.01, 0.10] | .029^*^ |
| **Short-term memory** | |  |  |  |  |  |  |
|  | Period | 0.08 | 0.04 | 2.12 | 1390.47 | [0.01, 0.16] | .035^*^ |
|  | Situational loneliness | 0.49 | 0.04 | 13.95 | 1391.16 | [0.42, 0.56] | < .001^***^ |
|  | Block order | 0.07 | 0.08 | 0.90 | 119.02 | [–0.09, 0.23] | .370 |
|  | Block order × Period | –0.22 | 0.06 | –3.89 | 1390.49 | [–0.33, –0.11] | < .001^***^ |
|  | Situational loneliness × Period | 0.06 | 0.03 | 2.26 | 1390.28 | [0.01, 0.12] | .024^*^ |
| **Working memory** | |  |  |  |  |  |  |
|  | Period | 0.03 | 0.05 | 0.58 | 1093.58 | [–0.06, 0.12] | .561 |
|  | Situational loneliness | 0.48 | 0.03 | 14.75 | 1094.19 | [0.42, 0.55] | < .001^***^ |
|  | Block order | 0.07 | 0.08 | 0.88 | 117.05 | [–0.09, 0.23] | .383 |
|  | Block order × Period | –0.22 | 0.07 | –3.30 | 1093.25 | [–0.36, –0.09] | < .001^***^ |
|  | Situational loneliness × Period | 0.07 | 0.03 | 2.15 | 1093.63 | [0.01, 0.14] | .031^*^ |
| **Executive function** | |  |  |  |  |  |  |
|  | Period | –0.08 | 0.04 | –1.94 | 1227.50 | [–0.16, 0.00] | .053 |
|  | Situational loneliness | 0.49 | 0.03 | 14.77 | 1228.33 | [0.42, 0.55] | < .001^***^ |
|  | Block order | 0.07 | 0.08 | 0.89 | 117.95 | [–0.09, 0.23] | .375 |
|  | Block order × Period | –0.34 | 0.06 | –5.72 | 1227.37 | [–0.46, –0.22] | < .001^***^ |
|  | Situational loneliness × Period | 0.01 | 0.03 | 0.20 | 1227.01 | [–0.05, 0.06] | .839 |

*Note.* *n* = 117 females. VmHRV is quantified using the root mean square of successive differences (RMSSD). vmHRV = vagally-mediated heart rate variability; LMM = linear mixed model; SE = standard error; CI = confidence interval. Period is coded 0 = baseline, 1 = task. Block order is coded 0 = first block, 1 = second block. ^*^ *p* < .05, ^**^ *p* < .01, ^***^ *p* < .001.

**Table S17.**

*LMMs Predicting vmHRV Reactivity to Cognitive Tasks from Situational Loneliness (Males Only)*

|  |  | β | SE | *t* | *df* | 95% CI | *p* |
| --- | --- | --- | --- | --- | --- | --- | --- |
| **Attention** | |  |  |  |  |  |  |
|  | Period | 0.09 | 0.07 | 1.27 | 364.05 | [–0.05, 0.23] | .207 |
|  | Situational loneliness | 0.16 | 0.06 | 2.69 | 364.35 | [ 0.04, 0.28] | .007^**^ |
|  | Block order | –0.14 | 0.12 | –1.11 | 33.57 | [–0.38, 0.10] | .274 |
|  | Block order × Period | –0.00 | 0.10 | –0.03 | 364.18 | [–0.20, 0.20] | .976 |
|  | Situational loneliness × Period | 0.02 | 0.05 | 0.34 | 364.37 | [–0.09, 0.12] | .737 |
| **Perception** | |  |  |  |  |  |  |
|  | Period | 0.12 | 0.05 | 2.25 | 433.02 | [ 0.02, 0.23] | .025^*^ |
|  | Situational loneliness | 0.16 | 0.05 | 3.17 | 433.20 | [ 0.06, 0.26] | .002^**^ |
|  | Block order | –0.14 | 0.12 | –1.14 | 33.47 | [–0.37, 0.10] | .263 |
|  | Block order × Period | 0.09 | 0.08 | 1.13 | 433.10 | [–0.06, 0.24] | .259 |
|  | Situational loneliness × Period | 0.05 | 0.04 | 1.15 | 433.23 | [ –0.03, 0.12] | .249 |
| **Short-term memory** | |  |  |  |  |  |  |
|  | Period | 0.05 | 0.06 | 0.79 | 394.21 | [–0.07, 0.17] | .430 |
|  | Situational loneliness | 0.16 | 0.05 | 3.04 | 394.23 | [0.06, 0.27] | .003^**^ |
|  | Block order | –0.14 | 0.12 | –1.17 | 33.50 | [–0.37, 0.09] | .252 |
|  | Block order × Period | 0.08 | 0.09 | 0.96 | 394.66 | [–0.09, 0.25] | .336 |
|  | Situational loneliness × Period | –0.01 | 0.05 | –0.23 | 394.13 | [–0.10, 0.08] | .819 |
| **Working memory** | |  |  |  |  |  |  |
|  | Period | –0.01 | 0.08 | –0.12 | 307.15 | [–0.16, 0.14] | .904 |
|  | Situational loneliness | 0.16 | 0.05 | 3.21 | 307.35 | [0.06, 0.26] | .001^**^ |
|  | Block order | –0.14 | 0.12 | –1.16 | 32.83 | [–0.38, 0.10] | .253 |
|  | Block order × Period | 0.07 | 0.11 | 0.69 | 307.11 | [–0.14, 0.29] | .490 |
|  | Situational loneliness × Period | –0.03 | 0.05 | –0.51 | 307.39 | [–0.14, 0.08] | .614 |
| **Executive function** | |  |  |  |  |  |  |
|  | Period | –0.07 | 0.07 | –1.02 | 331.18 | [–0.20, 0.06] | .309 |
|  | Situational loneliness | 0.17 | 0.05 | 3.22 | 331.19 | [0.07, 0.27] | .001^**^ |
|  | Block order | –0.14 | 0.12 | –1.17 | 32.99 | [–0.38, 0.10] | .250 |
|  | Block order × Period | –0.02 | 0.10 | –0.22 | 330.98 | [–0.21, 0.17] | .825 |
|  | Situational loneliness × Period | 0.07 | 0.05 | 1.46 | 331.53 | [–0.03, 0.17] | .146 |

*Note.* *n* = 34 males. VmHRV is quantified using the root mean square of successive differences (RMSSD). vmHRV = vagally-mediated heart rate variability; LMM = linear mixed model; SE = standard error; CI = confidence interval. Period is coded 0 = baseline, 1 = task. Block order is coded 0 = first block, 1 = second block. ^*^ *p* < .05, ^**^ *p* < .01, ^***^ *p* < .001.

**Table S18.**

*Linear Mixed Models Predicting HF-HRV Reactivity to Cognitive Tasks from Chronic Loneliness*

|  |  | β | SE | *t* | *df* | 95% CI | *p* |
| --- | --- | --- | --- | --- | --- | --- | --- |
| **Attention** | |  |  |  |  |  |  |
|  | Period | –0.10 | 0.04 | –2.72 | 1650.06 | [–0.17, –0.03] | .007^**^ |
|  | Chronic loneliness | 0.10 | 0.07 | 1.36 | 153.24 | [–0.04, 0.24] | .176 |
|  | Block order | 0.21 | 0.03 | 7.24 | 1651.48 | [0.15, 0.27] | <.001^***^ |
|  | Block order × Period | –0.04 | 0.05 | –0.73 | 1650.15 | [–0.14, 0.06] | .466 |
|  | Chronic loneliness × Period | 0.02 | 0.03 | 0.84 | 1650.06 | [–0.03, 0.07] | .400 |
| **Perception** | |  |  |  |  |  |  |
|  | Period | 0.16 | 0.03 | 5.49 | 1946.09 | [0.11, 0.22] | <.001^***^ |
|  | Chronic loneliness | 0.10 | 0.07 | 1.35 | 153.91 | [–0.04, 0.24] | .180 |
|  | Block order | 0.21 | 0.03 | 7.63 | 1947.14 | [0.16, 0.27] | <.001^***^ |
|  | Block order × Period | –0.10 | 0.04 | –2.42 | 1946.14 | [–0.19, –0.02] | .016^*^ |
|  | Chronic loneliness × Period | 0.03 | 0.02 | 1.32 | 1946.06 | [–0.01, 0.07] | .186 |
| **Short-term memory** | |  |  |  |  |  |  |
|  | Period | 0.02 | 0.04 | 0.47 | 1788.69 | [–0.05, 0.09] | .639 |
|  | Chronic Loneliness | 0.10 | 0.07 | 1.42 | 154.56 | [–0.04, 0.24] | .158 |
|  | Block order | 0.22 | 0.03 | 7.04 | 1789.48 | [0.16, 0.28] | <.001^***^ |
|  | Block order × Period | –0.05 | 0.05 | –0.96 | 1788.44 | [–0.15, 0.05] | .336 |
|  | Chronic loneliness × Period | 0.01 | 0.03 | 0.39 | 1788.82 | [–0.04, 0.06] | .699 |
| **Working memory** | |  |  |  |  |  |  |
|  | Period | –0.05 | 0.04 | –1.22 | 1404.87 | [–0.14, 0.03] | .222 |
|  | Chronic loneliness | 0.10 | 0.07 | 1.39 | 151.97 | [–0.04, 0.24] | .166 |
|  | Block order | 0.22 | 0.03 | 7.37 | 1405.76 | [0.16, 0.28] | <.001^***^ |
|  | Block order × Period | –0.07 | 0.06 | –1.10 | 1404.45 | [–0.19, 0.05] | .271 |
|  | Chronic loneliness × Period | 0.05 | 0.03 | 1.52 | 1405.11 | [–0.01, 0.11] | .130 |
| **Executive function** | |  |  |  |  |  |  |
|  | Period | –0.42 | 0.04 | –11.42 | 1562.74 | [–0.50, –0.35] | <.001^***^ |
|  | Chronic loneliness | 0.09 | 0.07 | 1.36 | 153.05 | [–0.04, 0.23] | .175 |
|  | Block order | 0.21 | 0.03 | 7.10 | 1563.73 | [0.15, 0.27] | <.001^***^ |
|  | Block order × Period | –0.07 | 0.05 | –1.24 | 1562.42 | [–0.17, 0.04] | .214 |
|  | Chronic loneliness × Period | 0.08 | 0.03 | 3.10 | 1563.11 | [0.03, 0.14] | .002^**^ |

*Note.* HF-HRV = high frequency heart rate variability (ln ms^2^). SE = standard error; CI = confidence interval.

Period is coded 0 = baseline, 1 = task. Block order is coded 0 = first block, 1 = second block. ^*^ *p* < .05, ^**^ *p* < .01, ^***^ *p* < .001.

**Table S19.**

*Linear Mixed Models Predicting HF-HRV Reactivity to Cognitive Tasks from Situational Loneliness*

|  |  | β | SE | *t* | *df* | 95% CI | *p* |
| --- | --- | --- | --- | --- | --- | --- | --- |
| **Attention** | |  |  |  |  |  |  |
|  | Period | –0.10 | 0.04 | –2.70 | 1650.05 | [–0.16, –0.03] | .007^**^ |
|  | Situational loneliness | –0.001 | 0.07 | –0.02 | 153.31 | [–0.14, 0.14] | .987 |
|  | Block order | 0.21 | 0.03 | 7.24 | 1651.45 | [0.15, 0.27] | <.001^***^ |
|  | Block order × Period | –0.04 | 0.05 | –0.76 | 1650.14 | [–0.14, 0.06] | .450 |
|  | Situational loneliness × Period | –0.01 | 0.03 | –0.40 | 1650.06 | [–0.06, 0.04] | .688 |
| **Perception** | |  |  |  |  |  |  |
|  | Period | 0.16 | 0.03 | 5.47 | 1946.09 | [0.11, 0.22] | <.001^***^ |
|  | Situational loneliness | –0.001 | 0.07 | –0.01 | 153.94 | [–0.14, 0.14] | .991 |
|  | Block order | 0.21 | 0.03 | 7.62 | 1947.11 | [0.16, 0.27] | <.001^***^ |
|  | Block order × Period | –0.10 | 0.04 | –2.40 | 1946.13 | [–0.19, –0.02] | .017^*^ |
|  | Situational loneliness × Period | 0.04 | 0.02 | 1.69 | 1946.22 | [–0.01, 0.08] | .091^†^ |
| **Short-term memory** | |  |  |  |  |  |  |
|  | Period | 0.01 | 0.04 | 0.42 | 1788.68 | [–0.05, 0.08] | .672 |
|  | Situational loneliness | –0.001 | 0.07 | –0.02 | 154.60 | [–0.14, 0.14] | .987 |
|  | Block order | 0.22 | 0.03 | 7.02 | 1789.45 | [0.16, 0.28] | <.001^***^ |
|  | Block order × Period | –0.04 | 0.05 | –0.89 | 1788.41 | [–0.14, 0.05] | .375 |
|  | Situational loneliness × Period | 0.06 | 0.02 | 2.38 | 1789.05 | [0.01, 0.11] | .017^*^ |
| **Working memory** | |  |  |  |  |  |  |
|  | Period | –0.05 | 0.04 | –1.21 | 1404.82 | [–0.14, 0.03] | .227 |
|  | Situational loneliness | –0.001 | 0.07 | –0.02 | 152.05 | [–0.14, 0.14] | .985 |
|  | Block order | 0.22 | 0.03 | 7.37 | 1405.73 | [0.16, 0.28] | <.001^***^ |
|  | Block order × Period | –0.07 | 0.06 | –1.09 | 1404.42 | [–0.19, 0.05] | .274 |
|  | Situational loneliness × Period | 0.03 | 0.03 | 0.80 | 1405.06 | [–0.04, 0.09] | .424 |
| **Executive function** | |  |  |  |  |  |  |
|  | Period | –0.42 | 0.04 | –11.32 | 1562.72 | [–0.50, –0.35] | <.001^***^ |
|  | Situational loneliness | –0.002 | 0.07 | –0.02 | 153.12 | [–0.14, 0.14] | .982 |
|  | Block order | 0.21 | 0.03 | 7.10 | 1563.69 | [0.15, 0.27] | <.001^***^ |
|  | Block order × Period | –0.07 | 0.05 | –1.25 | 1562.40 | [–0.17, 0.04] | .210 |
|  | Situational loneliness × Period | 0.03 | 0.03 | 1.10 | 1563.53 | [–0.02, 0.08] | .272 |

*Note.* HF-HRV = high frequency heart rate variability (ln ms^2^). SE = standard error; CI = confidence interval. Period is coded 0 = baseline, 1 = task. Block order is coded 0 = first block, 1 = second block. ^†^ *p* < .10, ^*^ *p* < .05, ^**^ *p* < .01, ^***^ *p* < .001.

| **Table S20.**  *Correlations between cognitive performance, vmHRV reactivity, and loneliness* | | | |
| --- | --- | --- | --- |
| Cognitive performance | vmHRV reactivity ^a^ | Chronic loneliness | Situational loneliness |
| Attention  *(visual search targets found)* | .02 | .02 | –.15 |
| Perception  *(visual comparison targets found)* | –.03 | –.18^*^ | –.13 |
| Short-term memory  *(forward digit span correct trials)* | .01 | .07 | .07 |
| Short-term memory  *(maximum forward digit span)* | .01 | .08 | .10 |
| Working memory  *(backward digit span correct trials)* | .01 | .05 | –.12 |
| Working memory  *(maximum backward digit span)* | –.02 | .02 | –.14 |
| Executive function  *(flanker task correct trials)* | .08 | .00 | .04 |
| *Note*. vmHRV = vagally mediated heart rate variability, quantified using the root mean square of successive differences (RMSSD). ^a^ vmHRV reactivity indicates the change in vmHRV from rest to the specific cognitive task listed, corresponding to the performance score with which it is correlated. ^*^ *p* < .05. | | | |

**Summary of HF-HRV (ln ms^2^) sensitivity analyses**

Separate linear mixed-effects models (LMMs) were specified for each cognitive task to test whether chronic loneliness predicted blunted HF-HRV (ln ms^2^) reactivity, and whether situational loneliness predicted either blunted or heightened HF-HRV reactivity. Each initial model included period (0 = initial rest, 1 = task), loneliness (chronic or situational), their interaction (loneliness × period), as well as block order (0 = first block, 1 = second block) and its interaction with period (block order × period). A random intercept for participant was specified in all models to account for between-person differences in resting HF-HRV (see Tables S18–S19 for full model outputs).

In all initial models, the effect of block order was significant suggesting overall HF-HRV was higher in the second block. With the exception of the perception task models, the block order by period interactions were not significant.

In the model examining the effect of chronic loneliness on HF-HRV reactivity during the executive function task, a significant effect of period indicated a general decrease in HF-HRV during executive function, β (SE) = –0.42 (0.04), *t* = –11.42, *p* < .001, 95% CI [–0.50, –0.35]. The interaction effect between chronic loneliness and period was also significant, suggesting those higher in chronic loneliness exhibited blunted HF-HRV reactivity (i.e., smaller HF-HRV decreases) during executive function demands, β (SE) = 0.08 (0.03), *t* = 3.10, *p* = .002, 95% CI [0.03, 0.14]. Although the other HF-HRV analyses for chronic loneliness revealed broadly similar directional patterns to the RMSSD analyses, the results of these analyses were weaker and did not reach statistical significance.

In the model examining the effect of situational loneliness on HF-HRV reactivity during the short-term memory task, the non-significant effect of period indicates overall HF-HRV during short-term memory did not significantly differ from overall HF-HRV during the rest period (*p* = .672). The interaction effect between situational loneliness and period was significant, suggesting those reporting higher situational loneliness demonstrated blunted HF-HRV reactivity (i.e., smaller HF-HRV decreases or larger HF-HRV increases) during short-term memory demands, β (SE) = 0.06 (0.02), *t* = 2.38, *p* = .017, 95% CI [0.01, 0.11].

**Figure S1.** Overview of the study procedure. The figure depicts the approximately 30-minute adaptation period followed by one 45-minute block of rest periods and tasks. Each participant completed two of these 45-minute blocks, with a break between blocks. The 10-minute filler task was a neutrally valenced task in which participants rated their impressions of images of faces displaying neutral expressions. The 15-minute cognitive task battery included five tasks administered in a fixed order, assessing attention, perception, short-term memory, working memory, and executive function. As part of a broader research project, each 45-minute block was administered under subtly different lighting conditions that were designed not to be visually apparent to participants, falling either outside the typical range of human visual detection or at its extreme edge. Lighting conditions were counterbalanced across participants. See the main text for details of the study procedure and the control of lighting condition and block order in analyses.

**Figure S2.** Predicted vagally mediated heart rate variability (vmHRV) reactivity to attention demands by levels of chronic loneliness, graphed separately for Block 1 and Block 2. VmHRV is quantified as the root mean square of successive differences (RMSSD), expressed in standardized units (z-scores). Lines depict estimated vmHRV trajectories from linear mixed-effects models at −1 SD, mean, and +1 SD of chronic loneliness (UCLA Loneliness Scale modified for a 5-year timeframe; Russell, 1996) to illustrate the significant association between higher chronic loneliness and blunted cardiac vagal withdrawal.

**Figure S3.** Predicted vagally mediated heart rate variability (vmHRV) reactivity to perception demands by levels of chronic loneliness, graphed separately for Block 1 and Block 2. VmHRV is quantified as the root mean square of successive differences (RMSSD), expressed in standardized units (z-scores). Lines depict estimated vmHRV trajectories from linear mixed-effects models at −1 SD, mean, and +1 SD of chronic loneliness (UCLA Loneliness Scale modified for a 5-year timeframe; Russell, 1996) to illustrate the significant association between higher chronic loneliness and blunted cardiac vagal withdrawal.

**Figure S4.** Predicted vagally mediated heart rate variability (vmHRV) reactivity to working memory demands by levels of chronic loneliness, graphed separately for Block 1 and Block 2. VmHRV is quantified as the root mean square of successive differences (RMSSD), expressed in standardized units (z-scores). Lines depict estimated vmHRV trajectories from linear mixed-effects models at −1 SD, mean, and +1 SD of chronic loneliness (UCLA Loneliness Scale modified for a 5-year timeframe; Russell, 1996) to illustrate the significant association between higher chronic loneliness and blunted cardiac vagal withdrawal.

**Figure S5.** Predicted vagally mediated heart rate variability (vmHRV) reactivity to executive function demands by levels of chronic loneliness, graphed separately for Block 1 and Block 2. VmHRV is quantified as the root mean square of successive differences (RMSSD), expressed in standardized units (z-scores). Lines depict estimated vmHRV trajectories from linear mixed-effects models at −1 SD, mean, and +1 SD of chronic loneliness (UCLA Loneliness Scale modified for a 5-year timeframe; Russell, 1996) to illustrate the significant association between higher chronic loneliness and blunted cardiac vagal withdrawal.
